# Exogenous human α-Synuclein acts in vitro as a mild platelet antiaggregant inhibiting α-thrombin-induced platelet activation

**DOI:** 10.1038/s41598-022-12886-y

**Published:** 2022-06-14

**Authors:** Laura Acquasaliente, Giulia Pontarollo, Claudia Maria Radu, Daniele Peterle, Ilaria Artusi, Anna Pagotto, Federico Uliana, Alessandro Negro, Paolo Simioni, Vincenzo De Filippis

**Affiliations:** 1 Laboratory of Protein Chemistry and Molecular Hematology, Department of Pharmaceutical and Pharmacological Sciences, School of Medicine, University of Padua, via Marzolo, 5, 35131 Padua, Italy; 2 Department of Women’s & Children’s Health, University of Padua, Padua, Italy; 3 Thrombotic and Hemorrhagic Diseases Unit, Department of Medicine, University of Padua, via Giustiniani, 2, 35128 Padua, Italy; 4 Department of Biomedical Sciences, University of Padua, viale G. Colombo 3, 35100 Padua, Italy; 5 Biotechnology Center, CRIBI, University of Padua, Padua, Italy; 6 Present Address: Center for Thrombosis and Hemostasis (CTH) University Medical Center Mainz, Langenbeckstraße 1, 55131 Mainz, Germany; 7 Present Address: Department of Chemistry and Chemical Biology, Northeastern University, 360 Huntington Ave. 02115, Boston, MA USA; 8 Present Address: Institute of Molecular Systems Biology, ETH Zurich, 8093 Zurich, Switzerland

**Keywords:** Proteases, Biochemistry, Neuroscience, Structural biology

## Abstract

α-Synuclein (αSyn) is a small disordered protein, highly conserved in vertebrates and involved in the pathogenesis of Parkinson’s disease (PD). Indeed, αSyn amyloid aggregates are present in the brain of patients with PD. Although the pathogenic role of αSyn is widely accepted, the physiological function of this protein remains elusive. Beyond the central nervous system, αSyn is expressed in hematopoietic tissue and blood, where platelets are a major cellular host of αSyn. Platelets play a key role in hemostasis and are potently activated by thrombin (αT) through the cleavage of protease-activated receptors. Furthermore, both αT and αSyn could be found in the same spatial environment, i.e. the platelet membrane, as αT binds to and activates platelets that can release αSyn from α-granules and microvesicles. Here, we investigated the possibility that exogenous αSyn could interfere with platelet activation induced by different agonists in vitro. Data obtained from distinct experimental techniques (i.e. multiple electrode aggregometry, rotational thromboelastometry, immunofluorescence microscopy, surface plasmon resonance, and steady-state fluorescence spectroscopy) on whole blood and platelet-rich plasma indicate that exogenous αSyn has mild platelet antiaggregating properties in vitro, acting as a negative regulator of αT-mediated platelet activation by preferentially inhibiting P-selectin expression on platelet surface. We have also shown that both exogenous and endogenous (i.e. cytoplasmic) αSyn preferentially bind to the outer surface of activated platelets. Starting from these findings, a coherent model of the antiplatelet function of αSyn is proposed.

## Introduction

**Figure 1:**
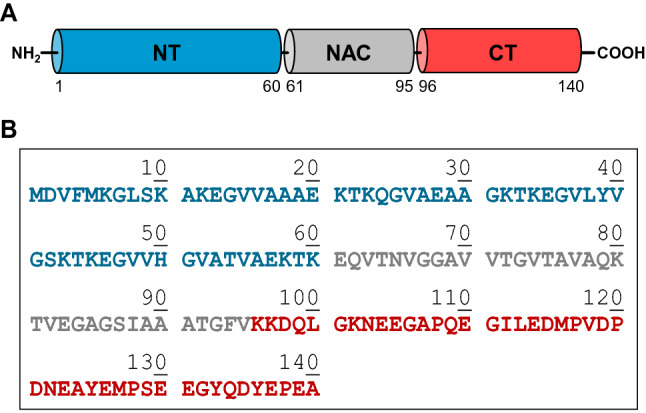
Domain architecture (**A**) and amino acid sequence (**B**) of human αSyn. NT: the N-Terminal domain region (amino acids 1–60) is positively charged (pI: 9.4) and assumes a helical conformation on lipid membrane surfaces. NAC: the Non-Amyloid α-Component (amino acids 61–95) is highly hydrophobic, has strong β-sheet conformational propensity, and mediates αSyn aggregation/fibrillation. CT: the C-Terminal domain (amino acids 96–140), is strongly negative (pI: 3.1).

α-Synuclein (αSyn) is a small acidic protein (140 amino acids; ~ 14 kDa) that is a highly conserved in vertebrates, and the presence of αSyn amyloid aggregates in the dopaminergic neurons of the brain *substantia nigra* is a key neuropathological hallmark of Parkinson’s disease (PD) 39^1,2^. αSyn is a structurally disordered monomeric protein, both when isolated in solution^3^ and in cellular environments, where it assumes a loosely packed dynamic structure^4^. The primary structure of αSyn displays three distinctive regions (Fig. 1): (i) the N-terminal region (NT, amino acids 1–60) is highly electropositive and serves to preferentially localize αSyn onto negatively charged biological membranes^5^; (ii) the central region, corresponding to the Non-Amyloid β-Component (NAC, amino acids 61–95) is hydrophobic in nature and crucial for fibrillation^6^; (iii) the C-terminal region (CT, amino acids 96–140) displays a high electronegative potential and is responsible for binding of αSyn to several target proteins^7^. Beyond a critical concentration (~ 30 μM) and after prolonged incubation in vitro, αSyn aggregates to form amyloid fibrils^8^, characterized by a cross-β-sheet structure^9^.

While the involvement of αSyn in the pathogenesis of PD is widely accepted, the normal physiological functions of this protein have yet to be fully elucidated^10^. αSyn is abundantly present in vivo in the human central nervous system^11,12^ and in the nuclei of neuronal cells and presynaptic terminals, where it binds to synaptic vesicles and modulates vesicle homeostasis and synaptic plasticity^11^. Beyond the central nervous system, significant expression levels of αSyn have also been measured in the hematopoietic tissue and blood (~ 1 μM; ~ 15 mg/l)^13–20^. The vast majority of blood αSyn is found in the erythrocytes (> 99%), as red blood cells are by far the most abundant haematologic cellular component, while the remaining amount is split between plasma (0.1%), leukocytes (0.05%) and platelets (0.2%). The latter are the main cellular hosts of αSyn in the blood^17^, containing 264 ± 36 ng of αSyn *per* mg of total proteins, twice as that stored in the erythrocytes (i.e. 131 ± 23 ng *per* mg of total proteins)^17^. αSyn-encoding mRNA was found in murine megakaryocytes^18^, while αSyn levels increase during differentiation of megakaryocytes into platelets^14^. Earlier cellular localization studies indicate that αSyn is abundantly present in the cytoplasm of resting platelets, associated with the secretory α-granules membrane, the inner leaflet of the plasma membrane^14,17,18^, and in platelet extracellular microvesicles^21^.

Upon vessel wall damage, platelets undergo a highly regulated set of functional responses, including: (i) adhesion, (ii) spreading, (iii) release reactions, (iv) aggregation, (v) exposure of a procoagulant surface, (vi) microparticle formation, and (vii) clot retraction^22–24^. All of these platelet responses cooperate to rapidly form the haemostatic plug at the site of vascular damage to prevent blood loss. More specifically, platelets play a central role in primary hemostasis, adhering to and being activated by subendothelial matrix proteins, such as collagen and von Willebrand factor (VWF) that become exposed after vascular injury^22^. VWF binding to the glycoprotein(Gp)Ib/IX/V complex on the platelet surface mediates initial platelet adhesion. Platelets then begin to slow down and transiently adhere to the vessel wall. Collagen binding to platelet GpVI results in cellular activation, followed by firm adhesion and spreading through the activated receptors GpIIb/IIIa and α_2_β_1_. Platelet adhesion also triggers intracellular signaling and platelet activation resulting into (i) degranulation, with the release (among others) of ADP, serotonin and Ca^2+^ from dense granules and VWF, fibrinogen, coagulation factors and the transmembrane glycoprotein P-selectin from α-granules, (ii) synthesis/release of thromboxane, (iii) activation of the GpIIb/IIIa complex on the platelet surface, (iv) exposure of anionic phosphatidylserine, and (v) generation of procoagulant microvesicles. Platelet activation then facilitates further local recruitment of platelets from the bloodstream, resulting in platelet aggregation mediated by fibrinogen and VWF bridging between activated GpIIb/IIIa on adjacent cells. The exposure of anionic phospholipid provides a negatively-charged surface upon which platelets can support coagulation factors complex assembly and activation (i.e. the tenase and prothrombinase complexes) during the amplification and propagation phases of α-thrombin (αT) generation and fibrin formation, resulting in the stabilization of the ensuing haemostatic plug^22^. Noteworthy, αT is the most potent platelet activator in vivo, cleaving the exodomain of two G-protein-coupled receptors (GPCR) on the platelet surface, i.e. type-1 and type-4 protease activated receptors (i.e. PAR1 and PAR4)^22^. αT also binds with high affinity to the α-chain of glycoprotein Ib (GpIb), a “leucine-rich repeat” receptor protein forming a non-covalent complex with GpV and GpIX on the platelet surface^25^. More specifically, the negatively charged C-terminal tail of GpIbα binds to the positively charged exosite 2 of αT^26^ and orients the protease exosite 1 for productive interaction with (and cleavage of) PAR1 exodomain. As a result, GpIbα enhances by > fivefold αT-induced proteolytic activation of PAR1^27^. After cleavage, the newly generated N-terminus acts as an intramolecular activator of PAR1, leading to degranulation and morphological and functional changes typical of activated platelets. Associated to these events is the exposure of the transmembrane glycoprotein P-selectin (CD62P) on the platelet surface, which is a signature of platelet activation mainly caused by αT and by non-proteolytic PAR1 agonists, such as TRAP6 (i.e. thrombin receptor activator peptide 6 corresponding to the N-terminal segment of the tethered PAR1 region SFLLRN-NH_2_)^28,29^. At variance with αT, adenosine-5′-diphosphate (ADP) weakly activates platelets by directly interacting with the P2Y_12_ receptor (i.e. the main GPCR for ADP on platelet membrane), reducing the concentration of cAMP and increasing cytosolic Ca^2+^, with final platelet shape change and activation^22^.

The plasma concentration of αSyn is significantly higher in patients with PD than in healthy individuals^30–35^ and abnormal platelet morphology (i.e., larger platelets) has been described in PD patients^19^, along with a decreased tendency of PD platelets to aggregate after stimulation with αT and ADP^36^. Interestingly, ischemic stroke, myocardial infarction, and coronary arterial disease appear to be significantly less frequent in patients with PD than in healthy controls^37–40^. Conversely, smaller platelets, increased platelet membrane expression of P-selectins, and a general hypercoagulable phenotype have been reported in α-syn-/- gene knockout mice, lacking αSyn^19,41^. Starting form this knowledge and considering that αT and αSyn can co-localize on the platelet surface (as αT binds to and activates platelets, which are also a major cellular reservoir of αSyn), we decided to investigate the in vitro effect of exogenous αSyn on the aggregation of platelets (both isolated and in whole blood) from healthy subjects, after stimulation with different agonists, i.e. αT, thrombin receptor activator peptide 6 (TRAP6), and ADP. Our results indicate that exogenous αSyn can act in vitro as a weak platelet antiaggregant, by mainly interfering with the αT-PAR1 functional axis. The possible physiological implications of these findings are also discussed.

## Results

### Production and characterization of recombinant αSyn species

Full-length human α-Synuclein (αSyn, amino acids 1–140), the corresponding N-terminal 6xHis-tagged derivative (6xHis-αSyn), the truncated species 6xHis-αSyn(1–96) and the fusion mutant protein αSyn-GFP, in which the polypeptide chain sequence of the Green-Fluorescent Protein (GFP) was fused with C-terminal end of αSyn, were expressed in *Escherichia coli* cells, as previously detailed^42,43^, and characterized for their purity and monomeric state, as reported in the Supplementary Fig. S1 and Table S1.

### Effect of αSyn and its derivatives on platelet activation/aggregation investigated by multiple electrode aggregometry, rotational thromboelastometry and flow cytometry

**Figure 2:**
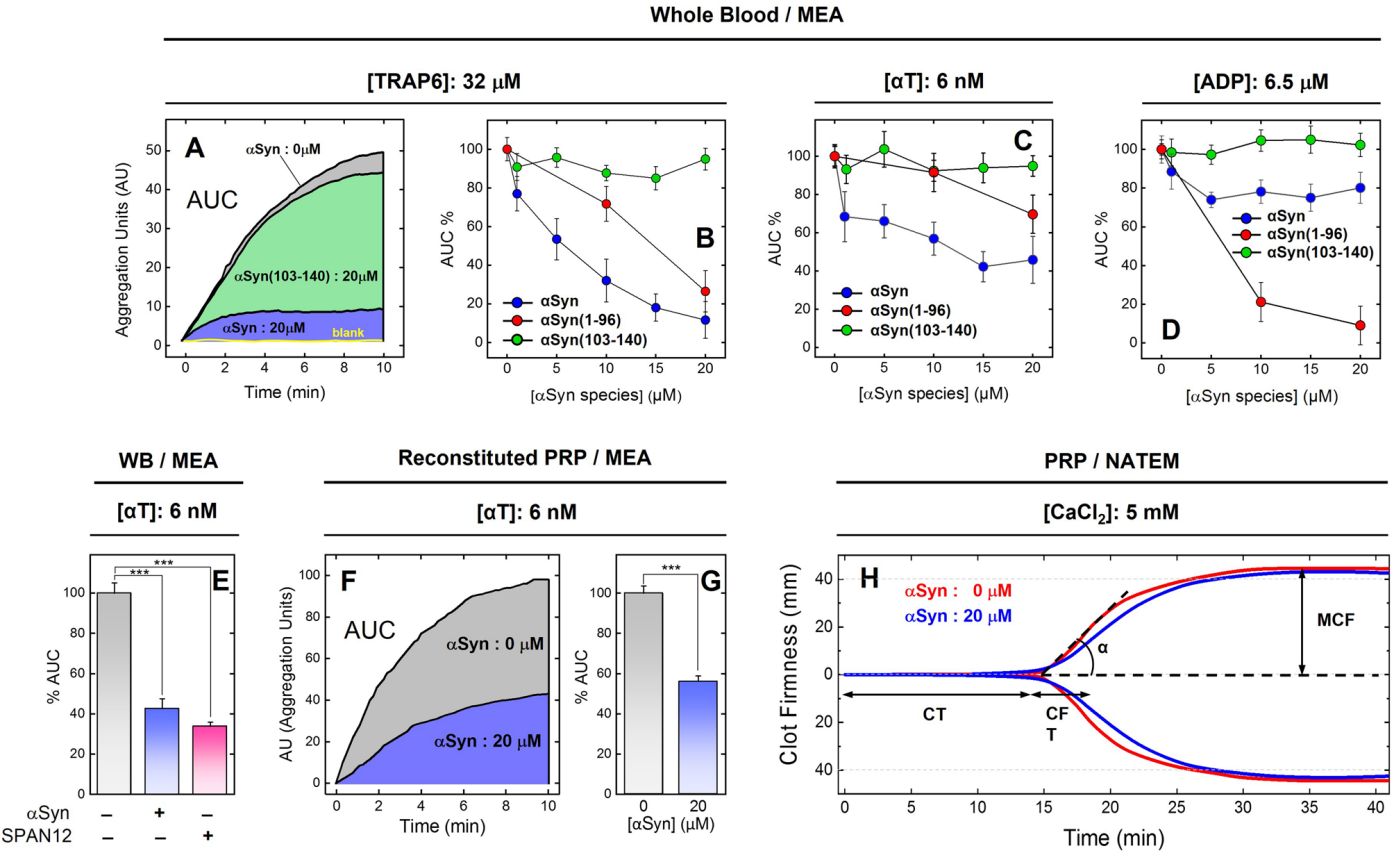
Effect of αSyn, αSyn(1–96) and αSyn(103–140) on the aggregation of platelets induced by TRAP6, αT or ADP. (**A**) Representative multiple electrode aggregometry (MEA) curves of platelets aggregation induced by TRAP6 on whole blood in the absence (grey area) and presence of 20 μM αSyn (blue area) or αSyn(103–140) (green area); blank experiment (yellow trace) without TRAP6 is also shown. (**B**–**D**) MEA analysis of platelet aggregation induced by 32 μM TRAP6 (**B**), 6 nM αT (**C**), and 6.5 μM ADP (D) on whole blood at 37 °C at increasing concentrations of full-length αSyn () αSyn(1–96) () or αSyn(103–140) (). At each αSyn concentration, the Area Under the Curve (%AUC) was calculated relative to the value determined in the absence of αSyn (AUC_0_). Whole blood samples from healthy donors (160·10^3^ platelets/μl) were incubated with solutions of αSyn species in HBS. Platelets aggregation was started by adding 20 μl of TRAP6 or ADP stock solutions, to reach the final agonist concentrations, as indicated. (**E**) MEA analysis of platelet aggregation induced by 6 nM αT in WB, in the absence (−) and presence (+) of 20 μM αSyn, and with or without 1 μM SPAN-12 antibody, as indicated. (**F**, **G**) αT-induced aggregation of isolated platelets in the presence of αSyn. (**F**) Representative MEA curves obtained at 37 °C after addition of αT (6 nM) to a reconstituted PRP sample containing 1 × 10^6^ platelets/μl (see Methods), in the absence (grey) and presence of 20 μM αSyn (blue). (**G**) Histogram plot of the platelets anti-aggregating effect of αSyn, as obtained for the data in panel F. For all MEA measurements, each AUC value is the average of single determinations on blood samples from three healthy donors, with error bars as ± SD. The height of the histograms is the mean value ± S.D. (n = 3). Significant differences were obtained from One-way ANOVA test and are indicated by lines joining the data being compared (***p < 0.001; **p < 0.01; *p < 0.05; ns: not significant). (**H**) Representative ROTEM analysis of PRP clotting in the absence (red trace) and presence (blue trace) of 20 μM αSyn, as indicated. Clotting was started by addition of 5 mM CaCl_2_. The values of CT, CFT, α, and MCF were extracted from three different measurements and reported as the mean value with ± SD. CT, − [αSyn]: 940 ± 50 s, + [αSyn]: 880 ± 40 s; CFT, − [αSyn]: 220 ± 15 s, + [αSyn]: 337 ± 20 s; α-angle, − [αSyn]: 52 ± 8°, + [αSyn]: 41 ± 6°; MCF, − [αSyn]: 45 ± 2 mm, + [αSyn]: 44 ± 2 mm. For details, see Methods.

*Multiple Electrode Aggregometry (MEA).* The effect of increasing concentrations of full-length αSyn and its N- and C-terminally truncated species on platelet aggregation was investigated by MEA analysis both with whole blood (WB) and washed platelets using TRAP6, αT or ADP as platelet activators (Fig. 2). MEA is a fast and specific platelet function test method^44^, widely used in basic hematologic research and clinical testing, allowing selective measurement of platelet aggregation not only in platelet-rich plasma (PRP) or isolated platelets, as with classical light transmission aggregometry (LTA), but also in WB, which is the physiological environment where platelet function takes place in vivo^45^ (see also “Methods”).

The data in Fig. 2A–D show that the addition of full-length αSyn to WB samples variably reduced, in a concentration-dependent manner, the platelet aggregation potential (%AUC) of all activators tested, where the strongest inhibitory effect was observed at 20 μM αSyn with TRAP6-induced activation (88 ± 9%), followed by αT (54 ± 12%) and ADP (21 ± 8%). An IC_50_ value of 8.6 ± 2.5 μM was estimated for the inhibition of αSyn in TRAP6-induced activation. As a negative control, the addition of αSyn to WB, in the absence of TRAP6, did not induce any change in AUC (Fig. 2A). In a different set of MEA experiments with WB, αSyn (20 μM) was found to inhibit platelet aggregation to an extent (56 ± 9%) comparable to that measured for a saturating concentration (1 μM) of SPAN-12 (65 ± 4%) (Fig. 2E), a monoclonal antibody selectively targeting the activation region (sequence 35–46) of PAR1 exodomain^46^. To rule out the possibility that other soluble and cellular components present in WB (e.g. fibrinogen, erythrocytes, and leukocytes) might interfere with platelet aggregation, MEA analysis was also performed with reconstituted PRP (see “Methods”) at the maximum concentration of αSyn explored with WB, i.e. 20 μM (Fig. 2F,G). Our data indicate that αSyn inhibits αT-induced platelet aggregation by 45 ± 5%, a value which is comparable with that obtained on WB at the same concentration (Fig. 2C). When the platelet antiaggregating activity of αSyn was tested with LTA in the αT-induced activation assay on PRP, a poor (if any) effect was detected (not shown).

To perform a structural dissection of the platelet antiaggregating function of αSyn, we performed in WB MEA analyses of the recombinant 6xHis-αSyn(1–96) polypeptide (pI 9.4), corresponding to the electropositive N-terminal region, and of the synthetic peptide αSyn(103–140) (pI: 3.1), encompassing the negative C-terminal tail of αSyn. 6xHis-αSyn(1–96) essentially retained the inhibitory effect of full-length αSyn on platelets activation by TRAP6, whereas the same polypeptide impaired αT-induced platelet activation to a lower extent compared to intact αSyn (Fig. 2B,C). Intriguingly, 6xHis-αSyn(1–96) appears to be a more potent inhibitor than αSyn in the ADP-test (Fig. 2D). It is noteworthy that the negatively charged αSyn(103–140) showed poor, if any, inhibitory potency in all platelet antiaggregating assays tested (Fig. 2B–D).

#### Rotational Thromboelastometry (ROTEM)

ROTEM^47^ analysis (Fig. 2H) was used to study the effect of 20 μM αSyn on the clotting kinetics of PRP, thus eliminating the possible effect of leukocytes and erythrocytes on clot formation. From the resulting TEMogram (i.e. the change of ROTEM signal vs. time) the clotting time (CT), the clot formation time (CFT), the α-angle value (α) and the maximal clot firmness (MCF) were extracted (see “Methods”). Our data indicate that addition of αSyn to PRP does not appreciably affect ROTEM parameters, except CFT and α-angle. In particular, CFT is prolonged on average from 220 ± 15 to 337 ± 20 s in the absence and presence of αSyn, respectively, whereas α is reduced on average from 52 ± 8 to 41 ± 6°.

#### Flow cytometry

**Figure 3:**
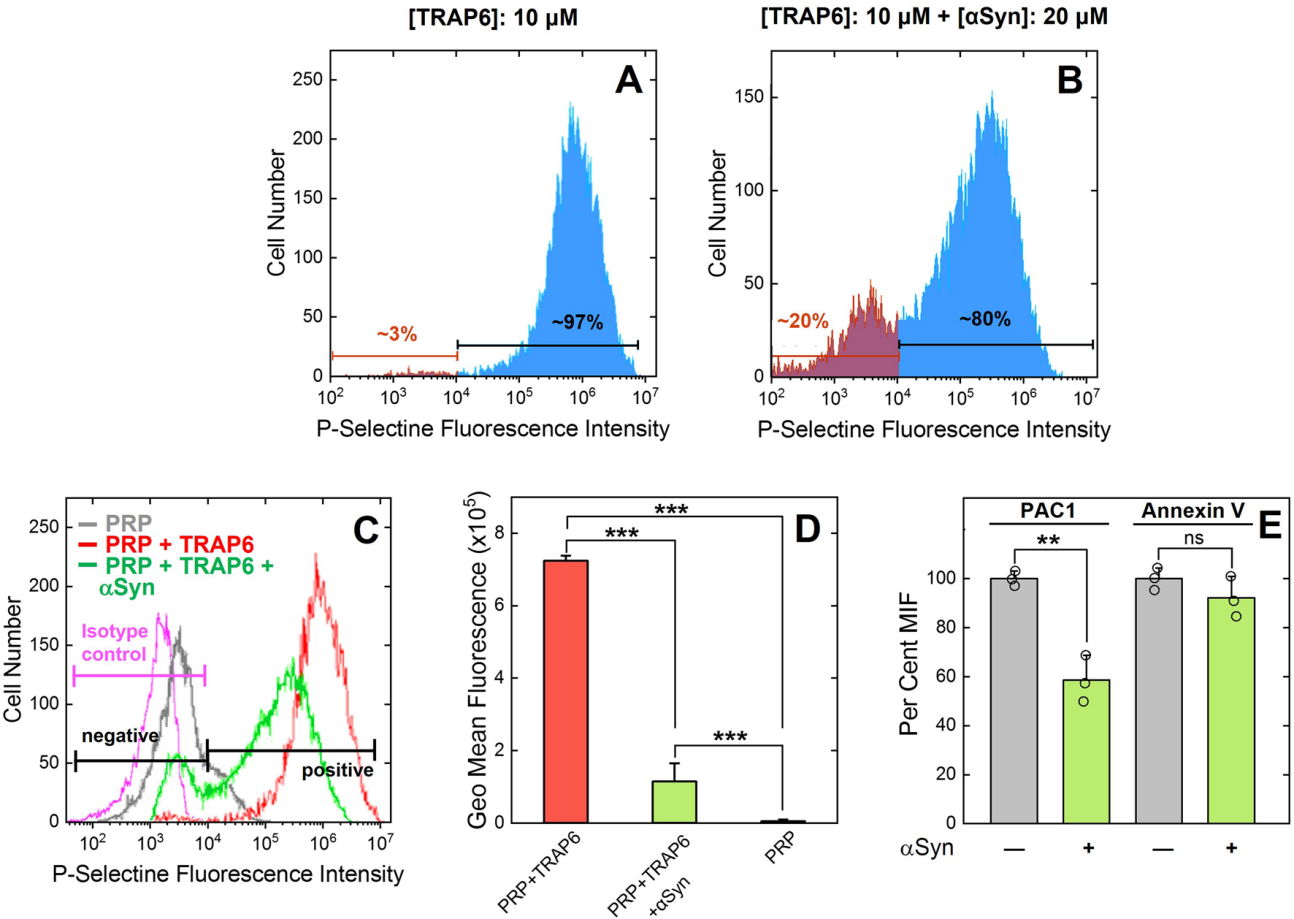
Flow Cytometry analysis of the effect of α-Syn on platelet activation after PAR1 stimulation with TRAP6. (**A**, **B**) Representative plots of P-selectin expression. P-selectin expression was detected using a phycoerythrin (PE)-conjugated monoclonal anti-human P-selectin (CD62P) antibody. A PRP sample (10 μl, 1 × 10^6^ platelets) was incubated with TRAP6 (10 μM) for 30 min at 37 °C, in the absence (**A**) and presence (**B**) of αSyn (20 μM). Fluorescence intensity was measured by flow cytometry after incubation of PRP with anti-CD62P-PE monoclonal antibody (20 min at r.t. in the dark). Values are reported as the mean relative abundance, resulting from three different determination, with errors (±) as S.D. (**C**) Overlay of the fluorescence intensity plots in panels A and B. The trace of PRP in the presence of anti-CD62P-PE antibody is shown in grey, while the negative isotype control with anti-mouse IgG1 PE-conjugated antibody is in magenta. The fluorescence intensity threshold, used to discriminate between activated (i.e. P-selectin positive) and non-activated (i.e. P-selectin negative) platelet subpopulations, was set at 10^4^ as indicated, and the % subpopulation was calculated by integration of the area under the curve (see “Methods”). (**D**) Quantitative GeoMean analysis of P-selectin (anti-CD62P-PE moAb) fluorescence intensities reported in panel (**C**). Data derive from three independent measurements (n = 3), with error bars ± SD. (**E**) Histogram plot of the mean fluorescence intensity (MFI) of FITC-labelled PAC-1 moAb, for the quantification of activated GP_IIb_/GP_IIIa_, and DY634-labelled Annexin-V, for the quantification of PS exposure. Data are extracted from the images in the Supplementary Fig. S3 and are reported as per cent values, relative to the MFI of TRAP6-stimulated PRP alone without αSyn, which was set at 100%. Data points are shown as empty circles (○), while the height of the histograms is the mean value ± SD (n = 3). Significant differences were obtained from One-way ANOVA test and are indicated by lines joining the data being compared (***p < 0.001; **p < 0.01; *p < 0.05; ns: not significant).

The ability of αSyn (20 μM) to inhibit platelet activation was investigated on PRP, after TRAP6-induced PAR1 stimulation, by monitoring the surface expression of P-selectin, activated GpIIb/IIIa, and PS^48^, using labelled monoclonal antibodies (moAb), as in the case of P-selectin (anti-CD62P-PE Ab) and GpIIb/IIIa (PAC-1 moAb), or labelled annexin V, a protein specifically recognizing surface exposed PS^48^. The homogeneity of platelet preparation (98 ± 2%) was established by the 2D scatter plot, representative of the forward and side scattering intensities of unlabeled platelets (not shown). When the same sample was challenged with anti-CD62P-PE-Ab, a small amount of platelets was found in the activated state (i.e. P-selectin positive), although with a low number of P-selectin molecules *per* platelet (gray trace, Fig. 3C). As estimated from the integration of the area under the curve (AUC) in Fig. 3A, after addition of 10 μM TRAP6 in the absence of αSyn, 97 ± 4% of platelets expressed P-selectin, while the remaining (3 ± 2%) were P-selectin negative. When PRP was preincubated with αSyn (20 μM) and then activated with TRAP6 (10 μM), a general reduction in the number of exposed P-selectin molecules *per* platelet cell was observed (Fig. 3B), along with a decrease in the percentage (%) of platelets in the activated state (i.e. P-selectin positive) from 97 ± 4 to 80 ± 3%, whereas the proportion of resting platelets (i.e. P-selectin negative) increased from 3 ± 2 up to 20 ± 2%(Fig. 3C). A quantitative analysis of the flow cytometry traces in Fig. 3C is reported in Fig. 3D. Next, we investigated the inhibitory effect of αSyn on the exposure of PS and activated GpIIb/IIIa (Fig. 3E). Although at the submaximal concentration of TRAP6 used in this study (i.e. 10 μM) only a small proportion of platelets were positive to PAC1 and Annexin-V (Supplementary Fig. S3), our data indicate that αSyn (20 μM) can significantly inhibit activated GpIIb/IIIa expression, whereas the effect on PS exposure is marginal.

### Probing αSyn platelet membrane localization by fluorescence microscopy

**Figure 4:**
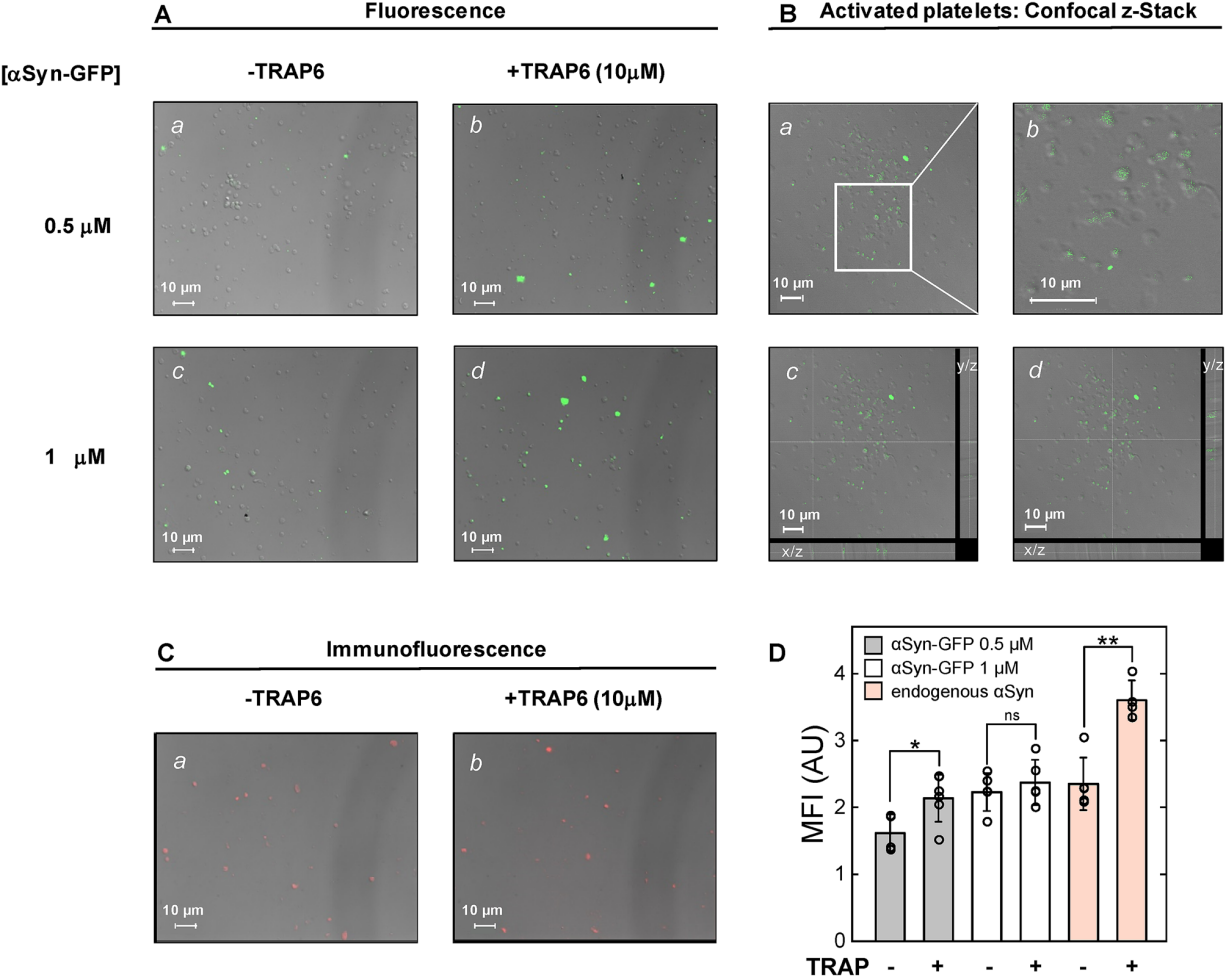
Fluorescence microscopy analysis of αSyn membrane localization in resting and activated platelets. Pictures result from merging fluorescence and differential interference contrast images (grey images) while platelet count was at 2 × 10^6^ platelets/well in all experiments. (**A**) Representative fluorescence microscopy images of exogenous αSyn binding to platelets. Isolated platelets were seeded, while increasing concentrations of αSyn-GFP (0, 0.5, and 1.0 μM) in HBS were added (1 h, 37 °C) to resting (− TRAP6) and activated (+ TRAP6) platelets, as indicated in panels *a*–*d*. After washing and paraformaldehyde fixation, seeded platelets were analysed by fluorescence microscopy. (**B**) panel *a*, Representative fluorescence confocal microscopy image of platelets activated with TRAP6 (10 μM) and then incubated (1 h, 37 °C) with exogenous αSyn-GFP (1 μM); panel *b*, Magnification of the rectangular area shown in panel *a*; panels *c* and *d*, Z-stack analysis of the fluorescence confocal image shown in panel *a*. Projections onto x/z and y/z planes are side views of serial confocal sections of the same area. (**C**) Representative immunofluorescence microscopy images of endogenous (i.e. cytoplasmic) platelet αSyn in resting and activated platelets. Resting (panel *a*) (− TRAP6) and activated (panel *b*) (+ TRAP6) platelets were seeded, fixed with paraformaldehyde and incubated (1 h, 37 °C) with mouse anti-human αSyn monoclonal antibody, followed by addition of a diluted Alexa Fluor 594-conjugated goat anti-mouse IgG (red fluorescence). Unspecific binding was assessed by incubating platelets with the secondary antibody alone. (**D**) Mean fluorescence intensity (MFI) values of exogenous αSyn-GFP and endogenous (i.e. cytoplasmic) αSyn bound to resting (− TRAP6) and activated (+ TRAP6) platelets. Data points are shown as empty circles (○), while the height of the histograms is the mean value ± SD (n = 4–6). Significant differences were obtained from Student’s t-test and are indicated by lines joining the data being compared (*p value < 0.05; **p value < 0.01; ns: not significant).

Images in Fig. 4A, D provide indication that exogenous αSyn-GFP at 0.5 μM binds to both resting and activated platelets with significant preference for activated platelets, compared to resting platelets. At higher αSyn concentration (1 μM), this binding preferentiality is greatly reduced, likely because at higher concentrations the plasma membrane of resting platelets approaches saturation with αSyn. The localization of αSyn-GFP was further investigated by fluorescence confocal microscopy and z-stack analysis (Fig. 4B), where orthogonal projections of the z-stack images on the *x/z* and *y/z* planes show the depth of the platelet section and strongly suggest the superficial binding of αSyn-GFP. Finally, using immunofluorescence microscopy and a highly specific anti-αSyn monoclonal antibody, we could also detect some basal exposure of endogenous αSyn even in resting platelets (Fig. 4C-a). Noteworthy, this exposure is significantly increased in TRAP6-activated platelets, compared to resting platelets (Fig. 4C-b and D).

### Effect of αSyn on fibrin generation

**Figure 5:**
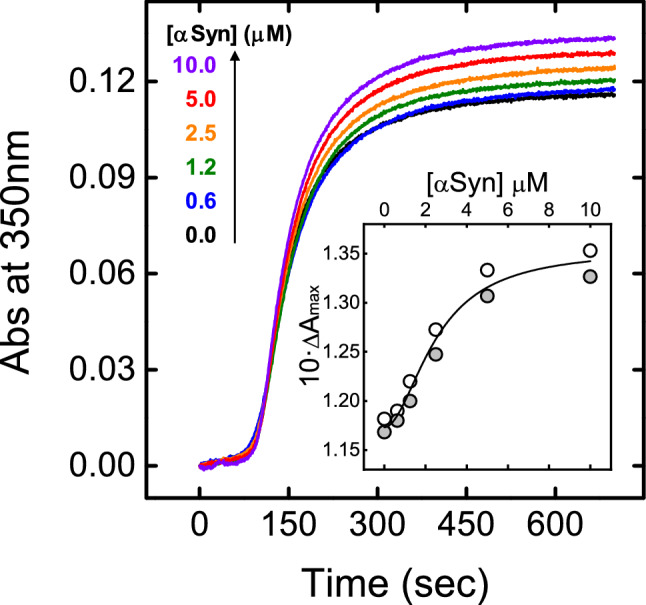
Effect of αSyn on αT-mediated fibrin generation. Representative turbidimetric analysis of fibrin generation induced by αT cleavage of purified fibrinogen at increasing αSyn concentrations, as indicated. To a desalted fibrinogen solution (440 nM, 800 μl) in HBS at 37 °C, containing 0.1% PEG-8000, was added αT (1 nM, final concentration) pre-incubated with increasing [αSyn] and the time-course of fibrin generation was monitored by recording the absorbance increase of the solution at 350 nm. From each clotting curve, the values of t_c_, and ΔA_max_ were extracted. (Inset) Plot of ΔA_max_ vs. αSyn concentration. Turbidimetric analysis was conducted in two independent measurements (n = 2), where each measurement was conducted as a technical duplicate. Each data point (○, ) represents the average of the corresponding technical duplicate.

The effect of αSyn on fibrin generation, from αT with purified fibrinogen, was monitored by turbidimetric analysis. The data shown in Fig. 5 indicate that, in the presence of αSyn, the clotting time (t_c_) remains essentially constant (109 ± 5 s), while the maximal change in turbidity (ΔA_max_) progressively increases up to 17 ± 2% at 10 μM αSyn (Fig. 5, Inset). These results suggest that αSyn does not alter the lag phase of fibrin formation, when longitudinal fibrin polymerization occurs, but appears to mainly perturb lateral aggregation to induce the formation of thicker fibrin fibers^49^.

### Effect of αSyn on αT-catalyzed substrate hydrolysis

**Figure 6:**
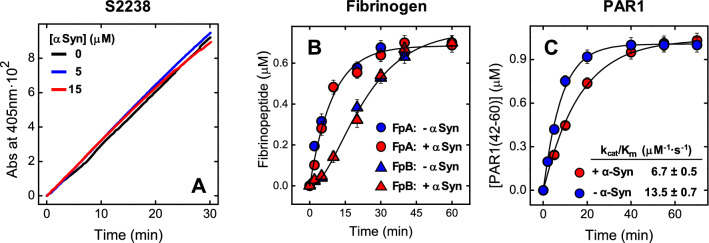
Effect of αSyn on αT-mediated hydrolysis of S2238, Fb and PAR1(38–60). (**A**) αT-catalyzed hydrolysis of the chromogenic substrate S2238 in the presence of increasing αSyn concentrations. The hydrolytic activity was determined at 37 °C in HBS by measuring the release of *p*-nitroaniline (*p*NA) at 405 nm. (**B**) Release of FpA and FpB from desalted fibrinogen (0.35 μM) by αT (300 pM), in the absence or presence of αSyn (15 μM). Measurements were carried out at 37 °C in HBS and quantified by RP-HPLC (see “Methods”). Interpolation of the data points with Eqs. 1 and 2 (Supplemenatry Material), yielded the apparent specificity constants (k_cat_/K_m_) of FpA and FpB release in the absence (k_cat,A_/K_m,A_ = 7.2 ± 0.9 µM^−1^·s^−1^; k_cat,B_/K_m,B_ = 5.7 ± 0.6 μM^−1^·s^−1^) and presence (k_cat,A_/K_m,A_ = 4.7 ± 2.2 μM^−1^·s^−1^; k_cat,B_/K_m,B_ = 4.6 ± 2.1 µM^−1^·s^−1^) of 15 μM αSyn. (**C**) Cleavage of PAR1(38–60) by αT in the absence and presence of αSyn (15 μM). The cleavage of PAR1(38–60) peptide (1 μM) by αT(150 pM) was carried out at 25 °C in TBS and the time course of PAR1(42–60) fragment release quantified by RP-HPLC. The data points were fitted with Eq. 3 (Supplemenatry Material), to yield the values of k_cat_/K_m_ in the absence and presence of αSyn. The data points are the average of three independent measurements (n = 3), with errors as ± SD.

The kinetics of *p*-nitroaniline release from the αT-specific substrate S2238 (Fig. 6A) clearly indicates that αSyn, up to the highest protein concentration explored (15 μM), does not significantly affect the rate of substrate hydrolysis. Identical results were obtained with the same concentration of αSyn(103–140) (not shown). Similarly, the release of fibrinopeptides (i.e. FpA and FpB) from a fibrinogen solution was not affected by 15 μM αSyn (Fig. 6B). Although αSyn (15 μM) was found to reduce by twofold the average efficiency of αT-catalyzed hydrolysis the synthetic peptide PAR1(38–60) encompassing the PAR1 activation domain, in energetic terms this effect (ΔΔG* = 0.41 kcal/mol) is lower than internal energy of an aqueous system at the same temperature (0.59 kcal/mol at 25 °C) and therefore it cannot be considered as significant (see Eq. 4 in the Supplementary Material)^50,51^ (Fig. 6C).

### Probing the αSyn-αT interaction by fluorescence spectroscopy and surface plasmon resonance (SPR)

**Figure 7:**
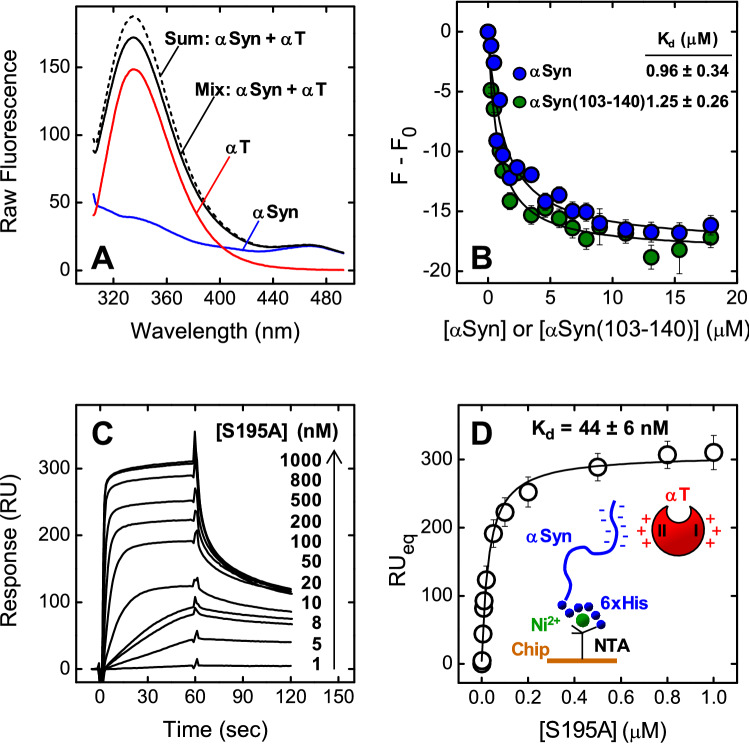
Probing αT-αSyn interaction by fluorescence spectroscopy and surface plasmon resonance. (**A**) Fluorescence spectra of isolated αSyn (20 μM) and αT (70 nM), and of the experimental complex containing the interacting proteins at the corresponding concentrations , as indicated. For comparison, the theoretical sum spectrum (--) is also reported. Emission spectra were recorded in HBS at 37 °C, after excitation at 295 nm, and subtracted for the corresponding baseline. (**B**) Fluorescence binding measurements of αSyn interaction with αT. To a solution of αT (70 nM) in HBS at 37 °C were added aliquots (2–20 μl) of full-length αSyn () and αSyn(103–140) (). The samples were excited at 295 nm, and the emission intensity was recorded at 334 nm. Each spectrum was subtracted for the contribution of αSyn alone at the corresponding concentration and expressed as F − F_0_, where F and F_0_ are the fluorescence intensity in the presence or absence of αSyn derivatives. The data points were interpolated with Eq. 5 (Supplementary Material), yielding the K_d_ value as indicated. (**C**, **D**) SPR analysis of αT binding to immobilized αSyn. Recombinant wild-type 6xHis-αSyn was immobilized onto a Ni^2+^-NTA sensor chip and increasing concentrations of S195A thrombin mutant were injected in the mobile phase. (**C**) Representative sensograms relative to the binding of S195A. (**D**) Plot of RU_max_ vs. S195A concentration (○). Fitting of data points with Eq. 8 (Supplementary Material) yielded the K_d_ value for the synuclein-thrombin complex, as indicated. All SPR measurements were carried out at 37 °C in HBS-EP^+^. The data points are the average of three independent measurements (n = 3), with errors as ± SD.

The first evidence of the formation of αSyn-αT complex came from the fluorescence emission spectra (Fig. 7A), obtained after excitation at 295 nm, indicating that the addition of αSyn (20 μM, final concentration) to a αT solution (70 nM, final concentration) reduced by 10 ± 1% the fluorescence intensity of the solution, compared to the theoretical sum spectrum of both isolated αT and αSyn at the same concentrations, without appreciably altering the wavelength of maximum emission (λ_max_ = 334 nm) of αT. A quantitative estimate of αSyn-αT interaction was obtained by recording the decrease of αT fluorescence at λ_max_ with increasing αSyn concentrations (Fig. 7B), to yield an equilibrium dissociation constant (K_d_) of 0.96 ± 0.34 μM. In SPR measurements, 6xHis-αSyn was non-covalently immobilized onto a Ni^2+^/nitrilotriacetate sensor chip and incremental concentrations of S195A were injected into the mobile phase (Fig. 7C, D). The catalytically inactive S195A thrombin mutant was used, as active αT was shown to cleave the fused 6xHis-αSyn at Lys6, but not the untagged wild-type αSyn 1–140 (Supplementary Fig. S2). Interpolation of SPR data allowed to estimate a K_d_ of 44 ± 6 nM. Notably, the affinity of αT for immobilized αSyn was estimated as > 20-fold higher than that determined by fluorescence binding experiments. This result is consistent with the notion that conformational ordering of a disordered protein (i.e. αSyn), after immobilization on the SPR sensor chip, results in a beneficial lower loss of binding entropy and, therefore, in a higher binding strength^52,53^.

### Molecular mapping of the αSyn-αT interaction

To identify the region of αSyn responsible for αT binding, we measured the affinity of αSyn(103–140) for αT by steady-state fluorescence (Fig. 7B). Our data indicate that αSyn(103–140) binds to αT with an affinity very similar (K_d_ = 1.25 ± 0.26 μM) to that of full-length αSyn (K_d_ = 0.96 ± 034 μM), suggesting that the negatively charged C-terminal tail of αSyn is likely the protein binding epitope for αT. Next, we mapped the sites on the αT structure that are involved in the interaction with αSyn. The active site and two positively charged patches, namely exosites 1 and 2, are the hot spots on αT responsible for the recognition of most physiological substrates and inhibitors^54–56^. The role played by these regions in binding to αSyn was assessed using “the site-specific perturbation approach”, previously exploited in our laboratory in the study of αT interactions^53,57–60^.

#### Active site

To check whether the active site region of αT is involved in αSyn binding, the affinity of ligands/inhibitors, having incremental size and mapping different αT subsites [i.e. *p*-aminobenzamidine (PABA)^61^, the chromogenic substrate S2238^57^, and hirudin fragment 1–47^59^] was measured in the absence and presence of 20 μM αSyn. The data in Supplementary Fig. S4 indicate that αSyn only marginally alters the affinity of all active site-specific ligands tested, suggesting that the αT catalytic site is not significantly involved in binding to αSyn. These data are consistent with the observation that αSyn has no effect on the hydrolysis rate of S2238 by αT (Fig. 6A).

#### Exosites 1 and 2

**Figure 8:**
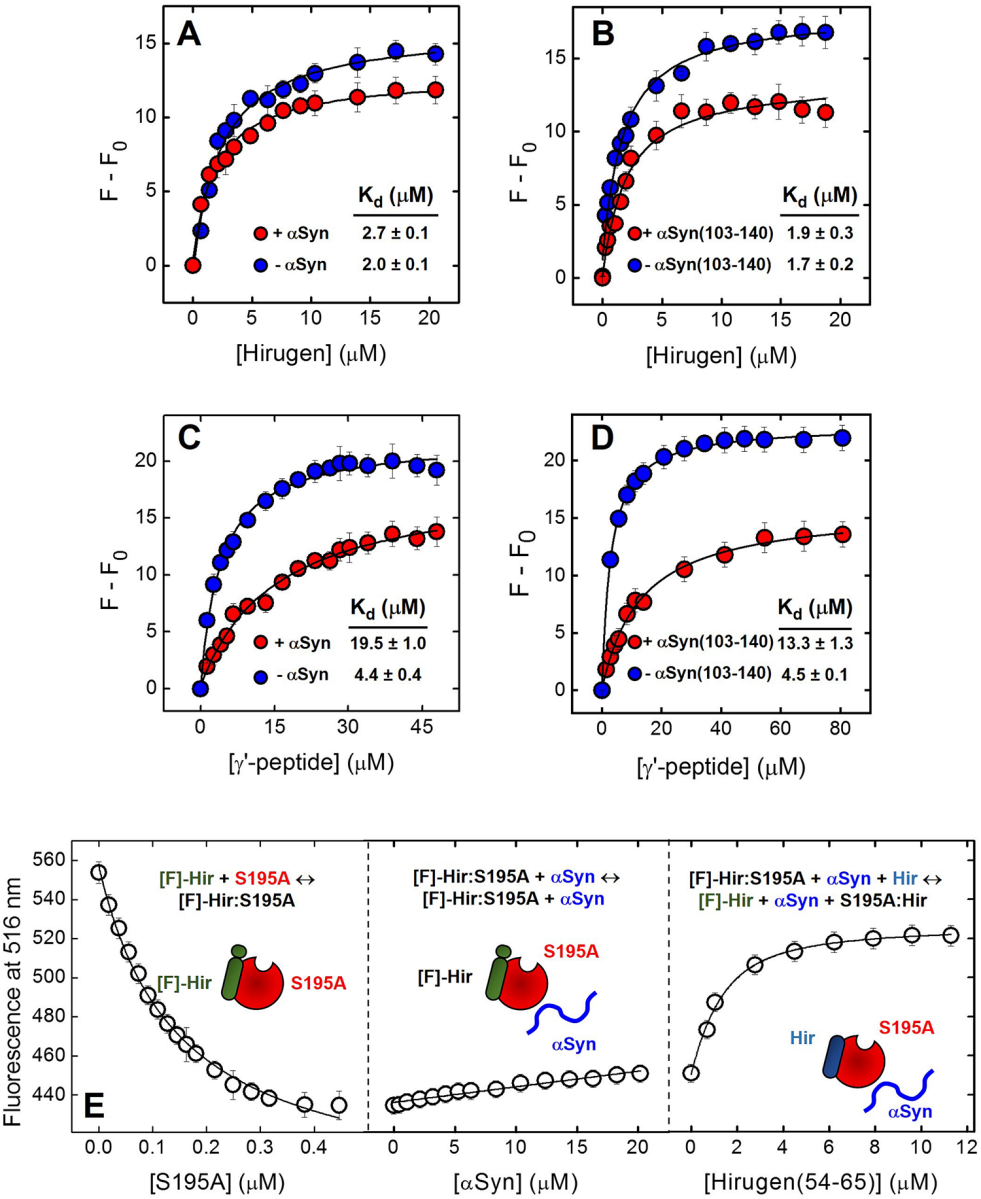
Probing the role of thrombin exosites in αSyn-αT interaction. (**A**, **B**) The role of exosite 1. Effect of αSyn and αSyn(103–140) on the affinity of hirugen for thrombin exosite-1. Fluorescence measurements of hirugen binding to αT (70 nM) in the absence or presence of saturating concentrations (20 μM) of full-length αSyn (**A**) or αSyn(103–140) (**B**). (**C**, **D**) The role of exosite 2. Effect of αSyn and αSyn(103–140) on the affinity of γ′-peptide for thrombin exosite-2. Fluorescence measurements of γ′-peptide binding to αT (70 nM) in the absence or presence of saturating concentrations (20 μM) of full-length αSyn (**C**) or αSyn(103–140) (**D**). Protein samples in HBS at 37 °C were excited at 295 nm and the fluorescence intensity was recorded at 334 nm, after baseline subtraction. The data points were interpolated with Eq. 5 (Supplementary Material) to obtain the K_d_ values, as indicated. (**E**) Displacement of [F]-hirugen from αT exosite 1 by αSyn. *Left panel,* Binding of S195A to [F]-hirugen (60 nM). The data points were interpolated with Eq. 6 (Supplementary Material), yielding a K_d_ of 30 ± 8 nM. *Middle panel*, Displacement of [F]-hirugen from the complex with S195A by incremental concentrations of αSyn. A moderate increase of fluorescence (~ 17%) was observed, suggestive of only a partial displacement of [F]-hirugen by αSyn. *Right panel*, Displacement of [F]-hirugen from S195A by incremental concentrations of unlabelled hirugen. A marked increase of fluorescence (~ 65%) was measured as the result of complete displacement of residual [F]-hirugen from αT exosite1. Samples in HBS, containing 0.1% PEG-8000, were excited at 25 °C at 492 nm and the fluorescence of [F]-hirugen, bound to or released from S195A, was recorded at 516 nm. All buffers contained 0.1% PEG-8000. The data points are the average of three independent measurements, with errors as ± SD.

The involvement of αT exosites in binding to αSyn was probed by fluorescence binding measurements, measuring the affinity of exosite-specific ligands for the protease in the absence and presence of αSyn or αSyn(103–140). Hirugen was selected as a safe exosite-1 binder^53,55^, while fibrinogen γ′-peptide was used as a specific exosite-2 ligand^62,63^. Full-length αSyn and αSyn(103–140) moderately reduced the affinity of the γ′-peptide for αT, on average by 4.4 and 3.0 times, respectively, as given by the K_d_ values in Fig. 8C, D. However, they were unable to impair hirugen binding at αT exosite 1 (Fig. 8A, B) nor displace N^α^-fluoresceinated hirugen ([F]-hirugen) from the same site (Fig. 8E).

**Figure 9:**
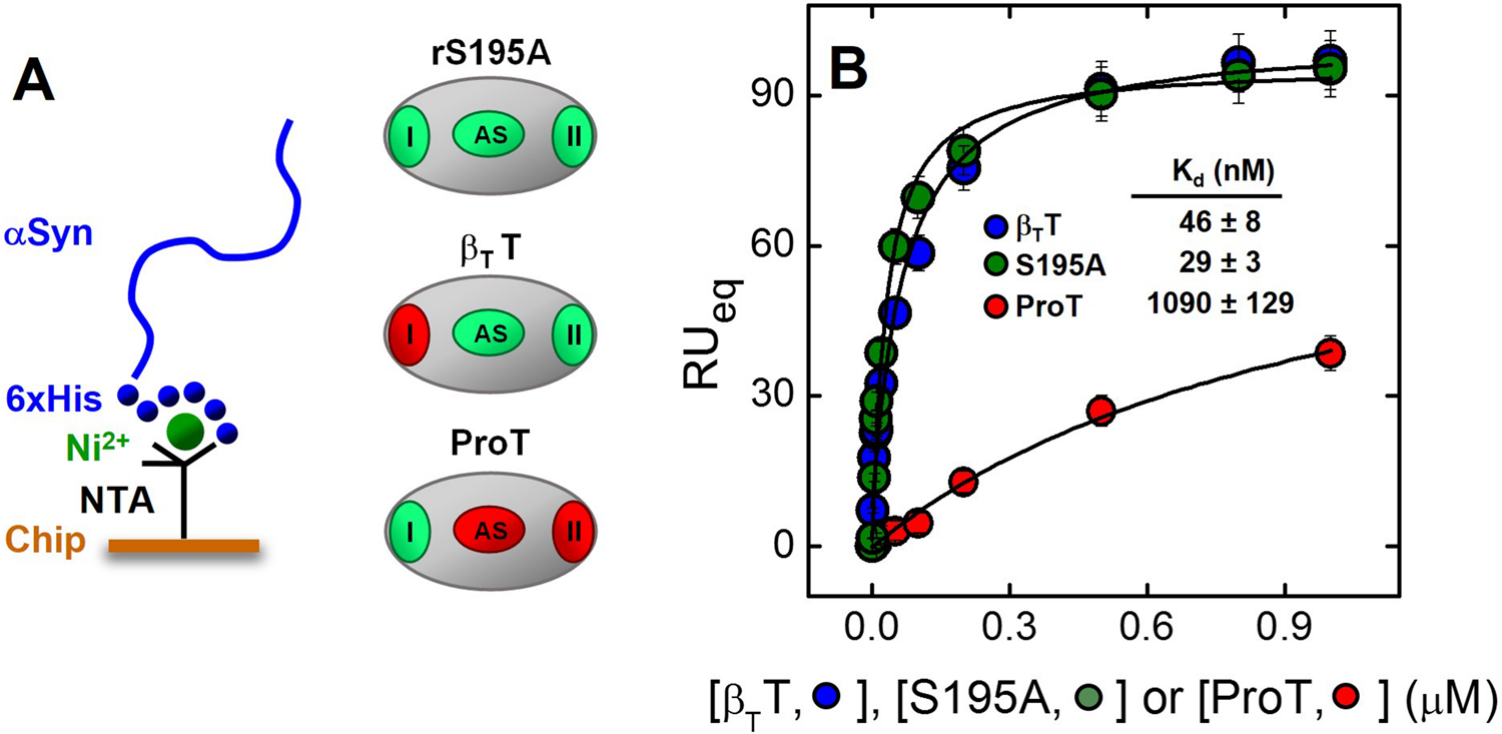
Effect of selective perturbation of thrombin exosites on the affinity for αSyn. (**A**) Schematic representation of the protease-domain of S195A thrombin mutant, β_T_-thrombin (β_T_T), and prothrombin (ProT). The active site (AS) and exosite 1 (I) and exosite 2 (II) are colored according to the conformational/functional state they assume in the different thrombin derivatives, compared to αT (see text); *green*: unperturbed or only slightly perturbed; *red*: heavily perturbed. (**B**) SPR analysis of the binding of S195A, β_T_T, and ProT to 6xHis-αSyn immobilized on a Ni^2+^-NTA sensor chip. The values of RU_max_ were plotted *versus* the concentration of thrombin derivatives and the data points were interpolated with Eq. 8 (Supplementary Material), yielding the corresponding K_d_ values as indicated. Measurements were carried out at 37 °C in HBS-EP^+^, pH 7.4. The data points are the average of three independent measurements, with errors as ± SD.

The role of αT exosites in the αT-αSyn interaction was further investigated by SPR, whereby the binding strength of thrombin species (i.e. β_T_T and ProT) to immobilized 6xHis-αSyn was measured (Fig. 9). β_T_T results from proteolytic nicking of mature αT by trypsin at the peptide bond Arg77a-Asn78, leading to disruption of exosite 1, while the active site and exosite 2 retain the structural and functional properties of the corresponding regions in mature αT^64^ (Fig. 9A). ProT is the physiological precursor of αT and, compared to αT, major structural perturbations occur at the Na^+^-binding site, the activation domain, and the insertion loops surrounding the catalytic cleft^59^. Importantly, the structure of exosite-1 appears to be only slightly altered in the structure of the zymogen, whereas the reactivity of exosite 2 is completely abolished due to intramolecular tight binding of the zymogen kringle-2 domain (Fig. 9A). Analysis of the binding data (Fig. 9B) shows that disruption of exosite 1, as in β_T_T, reduces the average affinity of the protease for 6xHis-αSyn by only 1.6 times, with a marginal difference in free energy change of binding (ΔΔG_b_ = 0.29 kcal/mole), lower than the internal energy at 37 °C (0.62 kcal/mol). Conversely, masking of exosite 2, as in ProT, leads to a dramatic drop in binding strength by 37 times, with a resulting significant ΔΔG_b_ = 2.2 kcal/mol (see Eq. 7 in the Supplementary Material).

Altogether, the results of molecular mapping indicate that αSyn uses its negative C-terminal tail 103–140 to preferentially interact with the electropositive αT exosite 2.

### Electrostatic properties of αSyn and its interaction partners

αSyn is a small acidic protein (pI 4.7) that, at physiological plasma pH, contains 16 positive and 25 negative charges, corresponding to 29.3% of its amino acid content and actually impairing αSyn to acquire a stable folded structure in solution^3^. The unique electrostatic properties of the protein are self-evident from its amino acid sequence (Fig. 1), since the N-terminal (NT) region (1–60) contains an excess of seven positive charges, while the C-terminal (CT) region (96–140) is strongly negative with an excess of 12 negatively charged amino acids. The central NAC region 61–95, which drives protein aggregation and fibrillogenesis, is highly hydrophobic and only slightly electropositive, with one positive charge in excess.

**Figure 10:**
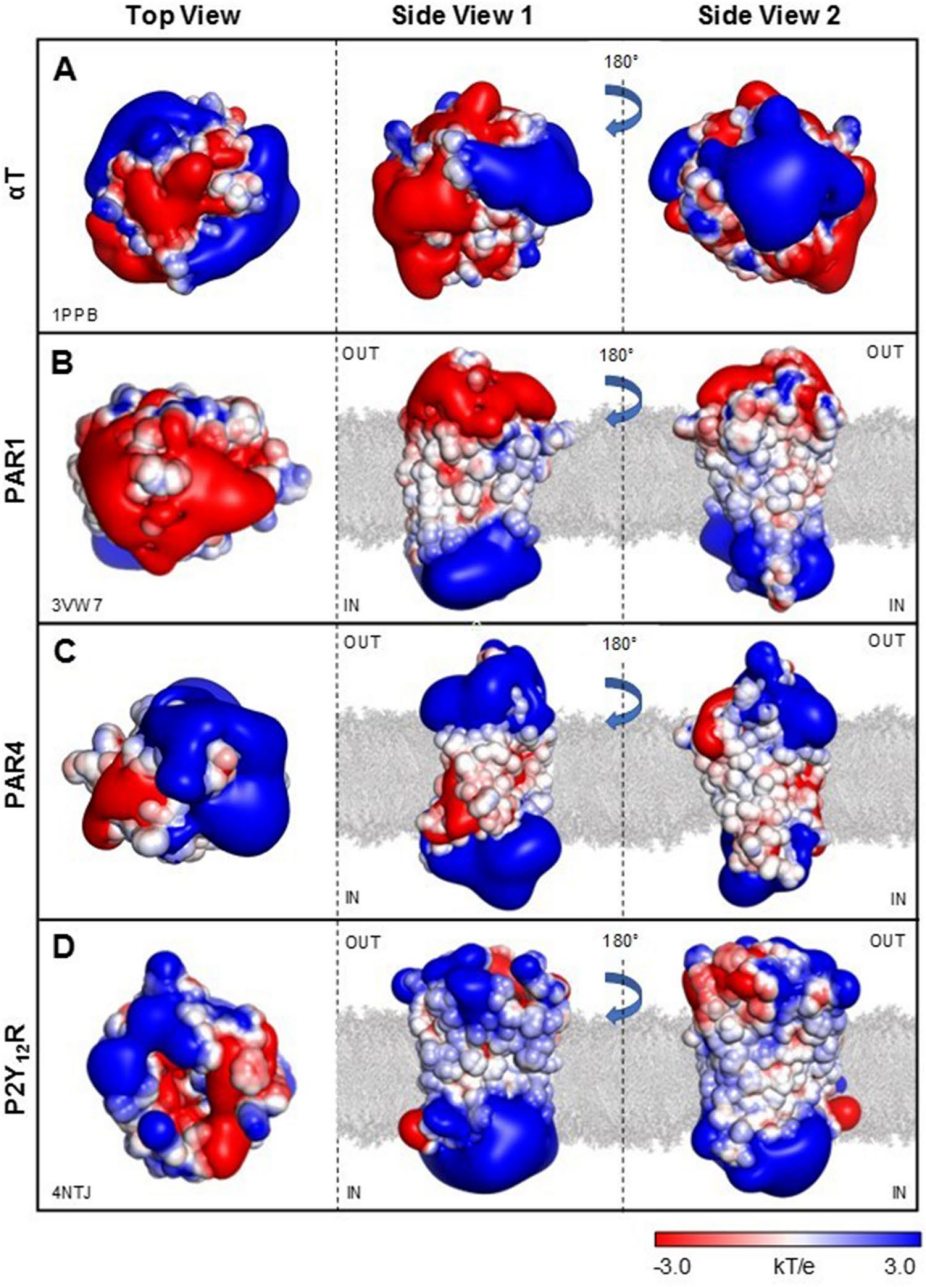
Surface electrostatic potential of αT (**A**), αT receptor PAR1 (**B**), and ADP receptor P2Y_12_R (**C**). Calculations were carried out using the APBS software on the deposited structures of αT (1 ppb), PAR1 (3vw7), P2Y_12_R (4ntj). PAR4 was modeled by homology on the PAR1 structure. Images were generated with PyMOL vs. 1.3 (DeLano Scientific, San Diego, CA, USA). The surface is coloured according to the electrostatic potential (blue, positive; red, negative) and expressed as kJ/(mol·q). Phospholipid double layer (grey) has been manually inserted. (**A**) αT displays an asymmetrical electrostatic potential, with a strongly negative active site (AS) region flanked by the two electropositive exosites (I and II). (**B**) The structure of PAR1, lacking the flexible exodomain A^22^-E^90^, displays a highly polarized charged distribution, with a strongly negative extracellular surface (OUT) and a positive intracellular region (IN). As expected, the transmembrane region in contact with the phospholipid apolar chains is essentially neutral. PAR1 contains a Na^+^-ion bound in the middle of the 7TM-helix bundle. The contribution of this ion was not taken into account during electrostatic calculations. (**C**, **D**) PAR4 and P2Y_12_R display a charge distribution similar to that of PAR1. However, contrary to PAR1, the extracellular region of P2Y_12_R and PAR4 is mainly electropositive with only some interspersed negative pots.

Even αT shows a non-uniform electrostatic potential (Fig. 10A), whereby exosites 1 and 2 are strongly electropositive, while the Na^+^ binding site and the active site region are negatively charged, with the catalytic pocket surrounded by a “negative ring” of Asp- and Glu residues^59^. Although both exosites display a positive electrostatic potential, exosite 2 is more electropositive and, indeed, ligand binding is mainly driven by “less specific” electrostatic charge-charge complementarity. At variance, beyond electrostatics, exosite 1 requires “more specific” interactions for binding^54–56^.

Electrostatic potential calculations (Fig. 10) indicate that the cytoplasmic region of all these receptors is always electropositive, while the extracellular region is strongly electronegative in PAR1 and substantially electropositive in both PAR4 and P2Y_12_R, with only some interspersed negative spots. As expected, the transmembrane regions in contact with the membrane phospholipids apolar chains are neutral in all the receptors investigated.

## Discussion

As an evolution of our previous work, aimed at identifying novel biochemical pathways at the interface of thrombosis and amyloidosis^53,60,65^, in this study we investigated the possible role of exogenous αSyn in platelet activation and found that αSyn functions in vitro as a mild platelet antiaggregating protein, acting primarily by inhibiting platelet PAR1 activation induced by TRAP6 (88 ± 9% inhibition) and αT (54 ± 12% inhibition), whereas ADP-induced activation is reduced by a lower extent (21 ± 8%) (Fig. 2A–D). When the inhibition of αSyn on αT-induced platelet aggregation in PRP was tested with LTA, only a small effect was detected (not shown). These differences can be only partially explained considering the reported higher sensitivity of MEA, compared to LTA, to small changes in platelet function^66–68^ and to the aggregating effect of αT^44,45^. In fact, MEA and LTA measure different aspects of platelet function^69^, as they use different sample material (WB vs. PRP), different measuring signals (changes in electrical impedance vs. changes in light transmission), and different shear rates (no shear vs. 1200 rpm). In LTA, platelet aggregation occurs in the liquid phase, a situation that presumably happens only in severely ill patients (e.g. during DIC), whereas MEA assay is performed in a biphasic system, where the increase in impedance of a blood sample is caused by sticking and intercellular adhesion of activated platelets onto the electrodes^44,45^. Most importantly, LTA is not influenced by interactions existing between platelets, red and white cells^69^, which instead have been shown to substantially modulate platelet aggregability in WB^70–73^, thus potentially affecting MEA response. On these grounds, it is not surprising that αSyn may cause different results in LTA and MEA assays.

The ability of αSyn to inhibit platelet aggregation induced by TRAP6, which is a direct PAR1 agonist, is compatible with a binding model in which αSyn interacts with PAR1 and competes with TRAP6 for receptor binding. On the other hand, the substantial residual thrombin activity, which is still observed in αT-induced platelet activation MEA measurements, could be explained considering that the protease, besides PAR1, also activates platelets via cleavage of PAR4^22^, which is not expected to be inhibited by the protein (see below). This interpretation is supported by the data in Fig. 2E, showing that after blocking PAR1 with a specific monoclonal antibody (SPAN-12), αT is still able to aggregate platelets, with a residual activity comparable to that observed with αSyn, in the absence of SPAN-12. At last, the small inhibitory effect observed with αSyn in the ADP test, more likely reflects an “unspecific sequestration” of the negative ADP^-3^ agonist by the highly electropositive αSyn N-terminal region rather than a “true” receptor inhibition. This interpretation is consistent with the known affinity of small/disordered electropositive nucleoproteins (e.g. histones) for (poly)nucleotides and with very recent data showing that protamine (51 amino acids, pI 12) antagonises ADP-induced activation of platelets^74^.

The possible effect of exogenous αSyn on the dynamics of the coagulation process was investigated by ROTEM, a viscoelastic technique allowing to simultaneously explore the time to clot formation and the clot strength^75,76^. Notably, the flex point in the TEMogram corresponds the maximal rate of αT generation^76^, while a prolongation of CT reflects an impaired function of the coagulative enzymes, and an increase in CFT, with a concomitant decrease of α indicates an alteration of fibrinogen cleavage/function. Although an increase of MCF is claimed to reflect enhanced platelet function and fibrin cross-linking^75^, recent clinical evidences show that this parameter is not adequate to assess the effects of most antiplatelet agents, as it remains constant with inhibitors of the thromboxane pathway (e.g. aspirin) and PAR1 (e.g. vorapaxar) and P2Y_12_ receptor (e.g. clopidogrel), and is reduced only with inhibitors of GpIIbIIIa (e.g. abciximab)^75,77^. ROTEM traces in Fig. 2G indicate that αSyn prolongs CFT and lowers the α-angle, while leaving CT and MCF essentially unchanged. These results indicate that αSyn acts as a mild inhibitor of clot formation and growth in the early stage of the coagulation process, mainly affecting fibrinogen activity, without significantly inhibiting the function of coagulative enzymes^75,76^.

A major achievement of this study entails the remarkable inibitory effect that exogenous αSyn has on TRAP6-induced exposure of P-selectins, along with the moderate inhibition of active GpIIbIIIa expression, as obtained by flow cytometry analysis. At variance, αSyn does not appreciably alter plasma membrane polarity and PS exposure (Fig. 3). Although the exposure of P-selectin, GpIIbIIIa and PS are all key biomarkers of platelet activation, they report on different activation pathways^31,48^. More specifically, P-selectin portrays α-granule release, whereas GpIIb/IIIa and PS are representative of the pro-aggregating and pro-coagulant potential of platelets, respectively (see “Introduction”). In quiescent platelets, P-selectins are located on the inner wall of α-granules and only after activation and α-granule secretion they are rapidly translocated to the platelet surface. Notably, P-selectin expression strongly correlates with the mean size of platelet aggregates and it is elevated in disorders associated with arterial thrombosis, where the involvement of platelets is even more important^28,72^. During platelet aggregation, GpIIb/IIIa undergoes activation-dependent conformational changes and becomes competent to bind soluble fibrinogen, which then cross-links platelets by bridging GpIIb/IIIa between adjacent platelets. After these initial events, P-selectin, progressively expressed on the platelet surface, stabilises interactions between already-bridged platelets by interacting with sulfatides on adjacent platelets, thereby allowing the formation of large stable platelet aggregates^28,72^. Furthermore, P-selectin expression promotes leukocyte recruitment to the site of thrombus formation, with a final pro-inflammatory effect that further boosts αT production and thrombus growth^71–73^. P-selectin, in fact, interacts with P-selectin glycoprotein ligand-1 on leukocytes and platelet GpIb, thus facilitating both leukocyte and platelet rolling and adhesion. P-selectin additionally triggers the release of procoagulant microvesicles and increases tissue factor expression on monocytes. Notably, inhibition of P-selectin by specific moAb leads to > 95% reversal of platelet aggregation, whereas a moAb against active GpIIb/IIIa (i.e. abciximab) displays no disaggregating effect^28,72^. For these reasons, P-selectin has become a major target for anti-aggregating drug development^79^. Hence, the remarkable inhibition of P-selectin exposure by αSyn, along with the moderate inhibitory effect on GpIIb/IIIa expression, can reasonably account for the anti-aggregating effect of the protein observed in MEA tests. Intriguingly, αSyn appears to have no (or negligible) effect on PS exposure. The latter result is consistent with ROTEM analysis, suggesting that αSyn does not appreciably interfere with the functions of the coagulative enzymes.

Another key aspect emerging from our work is that exogenous αSyn-GFP binds to the plasma membrane of platelets, with some preference for activated platelets compared to resting platelets, as given by z-stack fluorescence confocal microscopy (Fig. 4). Furthermore, localization studies, conducted by immunofluorescence microscopy, indicates that, after TRAP6 stimulation, endogenous (i.e. cytoplasmic) αSyn becomes significantly more exposed on the surface of activated platelets. The preferential localization of both exogenous and endogenous on the surface of activated platelets is in keeping with the reversal of plasma membrane charge polarization, occurring after platelet activation and leading to the exposure of negatively charged phosphatidylserine, and with previous in vitro studies showing that the positive N-terminal region drives binding of αSyn to negatively charged liposomes^5^ while the negative C-terminal tail fluctuates outside the membrane and remains largely unfolded^7^.

Besides the effects on PAR1-mediated platelet aggregation, our data also indicate that αSyn binds to αT with moderate to high affinity, as shown by steady-state fluorescence and SPR measurements (Fig. 7), without appreciably affecting the kinetics of αT-mediated fibrin generation and leading to the formation of thicker fibrin protofilaments (Fig. 5). These results are in keeping with the changes in CFT and α-angle observed in ROTEM analysis and can be explained considering that a highly charged protein, like αSyn, can interfere with longitudinal polymerization and lateral aggregation of fibrin, which are both variably influenced by charge-charge interactions^80^.

Next, we conducted a structural dissection of αSyn antiplatelet function (Fig. 2) and thrombin-binding properties (Figs. 8 and 9) by comparing the inhibitory effect of full-length αSyn in αT-, TRAP6- and ADP-test with that of the N-terminal region 1–96, which is highly positively charged, and with that of the C-terminal tail 103–140, which instead is strongly negative. Our results clearly indicate that the positively charged N-terminal region is mainly responsible for the platelet antiaggregating activity of αSyn in all assays tested, while the negative C-terminal tail is key for driving interaction of αSyn to the electropositive αT exosite 2. Noteworthy, αSyn(1–96) has a variable (and even opposite) effect on the platelets activation assays explored in this study. More specifically, in the TRAP6 test, αSyn(1–96) faithfully reproduces PAR1 inhibitory properties of intact αSyn, thus strongly supporting our hypothesis that the N-terminal region of αSyn could directly bind to and inhibit PAR1 activation. When tested in the αT-activation assay, αSyn(1–96) displays a reduced antiaggregating activity, compared to full-length αSyn, consistent with the lack of the C-terminal tail 103–140, which in the intact αSyn could bind αT on the platelet membrane and prevent protease binding to GpIbα and PAR1 activation. Surprisingly, the antiaggregant effect of αSyn(1–96) is increased in the ADP-activation test. As highlighted above for full-length αSyn, this effect is likely caused by the reduction in the effective concentration of ADP^-3^ agonist, which could be sequestered to a greater extent (compared to αSyn) by the highly electropositive αSyn(1–96) (pI: 9.4).

What emerges from our study is that antiplatelet activity and αT binding properties of αSyn is mainly dictated by the electrostatic properties of protein and those of its interacting partners (i.e. αT, PAR1, PAR4 and P2Y_12_R) (Fig. 10). PAR1 mainly exploits ligand-receptor electrostatic complementarity, which is less specific and mainly involves the superficial structures of the receptor^81^. On the contrary, ligand binding to P2Y_12_R^82^ and PAR4^83^ is more “stereochemically demanding” and it is driven by more specific orientation-dependent interactions, e.g. van der Waals interactions and hydrogen bonds. Therefore, in the case of αSyn(103–140), the lack of inhibitory activity toward platelet activation simply reflects the electrostatic repulsion of the electronegative peptide with the negative PAR1 exodomain and, likely, the poor stereochemical fit with PAR4 and P2Y_12_R. Similarly, the preferential inhibition of αSyn and αSyn(1–96) for TRAP6- and αT-induced PAR1 activation over ADP-induced P2Y_12_R activation, can be easily explained by the favorable electrostatic coupling of the positive N-terminal region of αSyn with the negative surface of PAR1 exodomain, whereas the same interaction with P2Y_12_R is hindered by electrostatic repulsion with the more positive receptor surface. Similar considerations can explain how 20 μM αSyn almost completely inhibits platelet activation by TRAP6 while, at the same concentration, activation by αT is inhibited only by 54 ± 12%. Electrostatics suggests that αSyn does not favorably interact with PAR4 and, therefore, the receptor could be still available for αT cleavage/activation. This interpretation is consistent with the data reported in Fig. 2E, showing that addition of αSyn (20 μM) or blockage of PAR1 with saturating SPAN-12 inhibit platelet aggregation by a comparable extent.

The experimental results reported in this study for exogenous αSyn, along with the preferential binding of both exogenous and endogenous αSyn for the negative surface of activated platelets, might serve to devise a model that accounts for the proposed mild platelet antiaggregating function of blood αSyn. Hence, we envisage that αSyn may function under physiological conditions as a negative regulator of αT-mediated platelet activation, acting either directly, through direct binding to PAR1, and indirectly, by interacting with platelets plasma membrane, where it could competitively bind αT and impair interaction of the protease with glycoprotein-Ibα and subsequent proteolytic activation of PAR1^27^. The αSyn modulatory function could be accomplished mainly through charge-charge interactions, acting long-range as an “electrostatic filter” to variably favor or disfavor binding to αT and platelet receptors. More specifically, cytoplasmic αSyn, secreted from α-granules after platelet stimulation, and freely circulating plasma αSyn can be effectively concentrated on the negatively charged plasma membrane of activated platelets through the positive NT region^5^, while the negative C-terminal tail remains disordered/flexible and available to: i) couple with the electropositive αT exosite 2, ii) bind/sequester the protease, and iii) down-regulate platelet activation. We speculate that this negative feedback mechanism could play a “protective” role against the platelet dysfunction and disintegration occurring after αT-induced activation^84^. In support of the antiplatelet function of αSyn, the lack of platelet αSyn in α-syn-/- gene knockout mice results in smaller platelets, increased platelet membrane expression of P-selectins, and a general hypercoagulable phenotype^19,41^.

Finally, the question arises of whether αSyn concentrations used in this study and proven to generate a mild antiaggregant effect in vitro could also have some relevance in vivo. The answer to this question is not easy to achieve, as αSyn is distributed in a complex heterogeneous system, between soluble (i.e. plasma) and cellular (i.e. erythrocytes, leukocytes and platelets) blood components^13–19^. Platelet αSyn is further split into a free cytoplasmic form and a bound form, the latter accumulating in α-granules^17^ and microvesicles^21^, from which it is secreted upon platelet activation and may even interact with the platelet plasma membrane. Further complications arise from the remarkable reduction (about threefold) of platelets volume after αT-stimulated activation^84^ and from the intrinsic difficulties in transforming αSyn concentrations of bulk solutions into membrane surface densities. In fact, at a given protein concentration in solution, the average distance between proteins in the membrane 2D space is shorter than in the solution 3D space^78^. This crowding effect increases the “density” of the membrane-bound proteins, with a consequent enhancement of the molecular interactions, which proceed more rapidly and to a greater extent on surfaces than in solution^85^. From the knowledge of platelet volume in the resting^86^ and activated^84^ states and αSyn concentrations in the plasma^17^ and within platelets^20^, using simple geometric parameters (see “Computational methods”), here we show that binding of exogenous (i.e. plasmatic) αSyn to activated platelets could produce an apparent “local” concentration of up to 1 μM, while secretion of endogenous (i.e. internal) αSyn and binding on the surface of activated platelets could result in an apparent αSyn “local” concentration of up to 20 μM, i.e. the maximal [αSyn] used in this work. Similar considerations may also apply to platelet-derived microvesicles, which are much smaller than platelets (d = 0.1–1 μm) and display a strong thrombotic potential^87^. From the knowledge of the average number of PAR1 molecules *per* resting platelet^88^, an apparent PAR1 concentration of 0.22 μM could be estimated (see “Computational methods”). Although the estimates of the “local” concentrations of αSyn and PAR1 reported above derive from coarse assumptions and simplifications, they are nevertheless plausibly in agreement with the functional role of αSyn proposed in this study.

In conclusion, the results reported in this study for exogenous αSyn suggest a novel function of blood αSyn, whereby the protein could act as a mild platelet antiaggregant, which unfolds outside the central nervous system, down-regulating αT-induced platelet activation. Our data suggest that aSyn inhibits platelet aggregation by mainly inhibiting PAR1-induced expression of P-selectin, playing a significant role in platelet aggregation and platelet-leukocyte interactions. Considered that platelets are a main source of αSyn, in severe thrombotic events, significant release of αSyn after platelet activation may have limiting effects on thrombosis propagation alongside with other endogenous inhibitory pathways (e.g. antithrombin-III, heparin-cofactor II, thrombomodulin-protein C). However, platelets are very complex systems and it is often difficult to precisely assign the effect of a given (exogenous) molecule to a change in the function of a specific biochemical pathway. In this view, further studies are needed to address the impact of αSyn in more complex models of platelet aggregation and blood coagulation both in vitro and in vivo.

## Methods

### Reagents

Human plasma αT (EC 3.4.21.5) and ProT were purchased from Haematologic Technologies (Essex Junction, VT, USA). Ecarin from *Echis carinatus* venom, bovine pancreatic trypsin, human plasma fibrinogen, Ac-Tyr-NH_2_, Ac-Phe-NH_2_, fluorescein isothiocyanate, and PABA were purchased from Sigma (St. Louis, MO, USA). The chromogenic substrate S2238 was from Chromogenix (Milan, Italy). SPAN12 monoclonal antibody was purchased from Beckman Coulter (Brea, CA, USA). Hirugen (^54^GDFEEIPEEY*LQ^65^) and [F]-hirugen^58^, fibrinogen γʹ–peptide (^408^VRPEHPAETEY*DSLY*PEDDL^427^)^63^, PAR1(38–60) (^38^LDPR↓SFLLRNPNDKYEPFWEDDE^60^)^57^, Hir(1–47)^51^, and αSyn(103–140) were synthesized by standard solid phase N^α^-fluorenylmethyloxycarbonyl chemistry on a PS3 automated synthesizer (Protein Technologies Int., Tucson, AZ, USA), purified to homogeneity (> 98%) by semipreparative RP-HPLC, and characterized by high resolution mass spectrometry. Notably, Y* indicates phosphorylated Tyr residues. N^α^-Fmoc-protected amino acids, solvents, and reagents for peptide synthesis were purchased from Applied Biosystems (Forster City, CA, USA) or Bachem AG (Bubendorf, Switzerland). Salts, solvents, and other reagents were of analytical grade and purchased from Sigma or Fluka (Darmstadt, Germany).

### Production and characterization of recombinant αSyn derivatives

All recombinant human synuclein derivatives (i.e. αSyn, 6xHis-αSyn, 6xHis-αSyn(1–96), and αSyn-GFP), were produced and purified as previously described^42,43^. For details, see the Supplementary Material. Freshly dissolved αSyn samples were used for further spectroscopic and functional analyses. The purified αSyn solutions were divided into aliquots, lyophilized, and stored at − 20 °C. After thawing in an ice-water bath, αSyn aliquots were immediately used for subsequent functional/binding analyses.

### Production and characterization of thrombin derivatives

The plasmid containing prethrombin-2 cDNA was a generous gift from Prof. James A. Huntington (University of Cambridge, Cambridge, UK). The inactive recombinant mutant S195A, obtained by single-point mutagenesis, was expressed in *E. coli*, subjected to in vitro disulphide oxidative folding, activation by ecarin, and characterized as previously detailed^57,60^. β_T_-thrombin (β_T_T) was obtained by proteolysis of human αT (7 μM) with bovine pancreas trypsin (35 nM) for 3 h at 37 °C in HBS, i.e. 4-(2-hydroxyethyl)-1-piperazineethanesulfonic acid (HEPES) buffered saline, pH 7.4, and characterized as described elsewhere^53,58^.

### Blood handling and preparation of PRP/PPP/reconstituted PRP

For MEA analyses on whole blood (WB), citrate-treated (3.2% buffered citrate in a 1 citrate: 9 WB proportion) venous blood samples were taken from five healthy donors: two men and three women, 28–35 years of age, and nonsmokers. Freshly withdrawn blood samples were used in all analyses. Donors gave their written informed consent to participate in this study, which was approved by the Institutional Ethics Committee of the Padua University Hospital and all methods were performed in accordance with the relevant guidelines and regulations. Platelet rich (PRP) and platelet poor (PPP) plasma samples were obtained after centrifugation of WB at 250 g for 10 min (PRP), or at 1500 g for 15 min (PPP), at r.t. without brake^89,90^. The supernatant was collected and used for further analysis. Leukocyte contamination (< 2%) of PRP samples was estimated by flow cytometry, using the leukocyte-specific CD45 marker kit (Abcam, Cambridge, UK). Before use, WB and PRP samples were recalcified with a 200 mM CaCl_2_ stock solution to a final concentration of 5 mM. Isolated/washed platelets were obtained after dilution (5:1, v/v) of PRP with PBS, pH 7.4, 10 mM EDTA, and centrifugation at 12.000 g (1 min, at r.t.) to allow platelet sedimentation. The pellet was washed twice with PBS/EDTA and finally resuspended with HBS, pH 7.4, or with autologous PPP to prepare a reconstituted PRP sample (rPRP), containing the desired platelet counts (1 × 10^6^ platelets/μl). Platelet counts were determined with a Cell-Dyn Emerald 22 haematology analyzer cytometer (Abbott Diagnostics, Chicago, IL, USA).

### Multiple electrode aggregometry (MEA) and light transmission aggregometry (LTA)

The effect of αSyn species on platelet aggregation induced by αT, TRAP, or ADP was measured at 37 °C by MEA in WB and with rPRP, using a Multiplate analyzer (Dynabyte, Munich, Germany)^45^. The physical basis of MEA relays on the increase of impedance (i.e. the electric resistance to the passage of alternate current in a medium between two platinum electrodes) that is caused by sticking and subsequent intercellular adhesion of activated platelets onto the electrodes^44,45^. Quiescent platelets adhere to the electrodes and self-organize in cell monolayers. At this stage, platelet-electrode interaction does not increase the impedance signal. Only after the addition of a platelet aggregating agent, activated platelets adhere tightly to the preexisting monolayers on the electrodes, thus increasing the electric impedance of blood. Increasing concentrations (0–20 μM; 300 μl in HBS) of monomeric samples of αSyn species were preincubated (30 min, 37 °C) with WB (300 μl, 160.000–200.000 platelets/μl) or rPRP (1 × 10^6^ platelets/μl). Platelet aggregation was started by adding TRAP6 or ADP stock solutions (20 μl) and MEA measurements were performed for 10 min. ADP-test and TRAP-test solutions for Multiplate assays were purchased from Roche Diagnostics (Basel, Switzerland). When the effect of αSyn species on αT-induced aggregation was measured, protease solutions (20 μl in HBS) were preincubated (30 min, 37 °C) with increasing concentrations of αSyn (0–20 μM, 300 μl in HBS) and then added to blood or PRP samples (300 μl). To obtain a quantitative estimate of platelet aggregation, the time-dependent change in blood impedance is expressed as relative Aggregation Units (AU), where 8 AU approximately correspond to 1 Ohm (Ω). Integration of AU over time gives the value of the Area Under the Curve (AUC), which is taken as the best parameter of platelet function in MEA analysis^45^. For each MEA measurement, the AUC was determined for single donors and the average value expressed as %AUC, relative to the value determined in the absence of αSyn (AUC_0_)^57,60^. LTA measurements were performed using a Chrono-Log (Havertown, PA, USA) model 700 aggregometer. PRP (500 μl, 350·10^3^ platelets/μl) was incubated with increasing concentrations of αSyn (0, 2, 5, 10, 20 μM) for 5 min at 37 °C, under continuous stirring (1200 rpm). Platelet activation was induced by adding 10 μM TRAP6 or 2 nM αT. Measurements were conducted in the Optical operation mode, with a luminescence gain of 0.005.

### Rotational thromboelastometry (ROTEM)

The effects of αSyn on PRP clotting were estimated using a ROTEM Delta analyzer (Instrumentation Laboratory, Milan, Italy) as previously described^91^*.* ROTEM measures the amount of a rotational force continuously applied that is transmitted to an electromechanical transduction system during clot formation and growth. PRP was allowed to clot without the addition of any activator other than calcium (i.e. NATEM, native ROTEM), in the absence and presence of αSyn. To avoid interindividual variability, which still limits the application of ROTEM in large-scale clinical testing, triplicate measurements were performed on a pool of PRP samples taken from three healthy subjects (two men and one woman, 35–50 years old) and the data are reported as the mean values with standard deviation ( ±). PRP samples (300 μl, 6 × 10^6^ platelets) were added with 20 μl of a αSyn stock solution (340 μM) in PBS, or with 20 μl of PBS alone for the blank experiment. According to the manufacturer’s procedures, the samples were incubated at 37 °C for 30 min on the ROTEM instrument prior to analysis. ROTEM analyses under native conditions (i.e. NATEM) were started by addition of a CaCl_2_ solution (final concentration 5 mM). Data were collected for 60 min and the coagulation parameters of clot formation (see legend to Fig. 2) were extracted from the experimental TEMograms using ROTEM-Delta software: clotting time (CT), the time from the beginning of the reaction to an increase in amplitude of thromboelastogram of 2 mm; clot formation time (CFT), the time in seconds between an increase in amplitude from 2 to 20 mm; α-angle, the slope of the tangent to the clotting curve through the 2-mm point; maximal clot firmness (MCF), the maximum amplitude in mm reached in the thromboelastogram.

### Flow cytometry analysis

Briefly, PRP samples (10 µl, 1 × 10^6^ platelets) were incubated with 5 μl of the proper labelled antibody or Annexin V. Platelets were analysed in the resting state and after activation with 10 μM TRAP6, in the absence and presence of αSyn (20 μM). αSyn was incubated for 30 min at 37 °C with PRP prior to analysis. Samples were then incubated with labelled antibodies or annexin V for 20 min at r.t. in the dark. For P-selectin (CD62P) detection, a phycoerythrin(PE)-conjugated monoclonal anti-human P-selectin antibody (anti-CD62P-PE-Ab) was used (Beckman Coulter, Miami, FL, USA). For PS exposure, the Annexin-V DY-634 kit was used (Abcam, Cambridge, UK). For GPIIb/IIIa detection, fluorescein isothiocyanate(FITC)-conjugated PAC-1 (Thermo Fischer Scientific) was used. As a negative control, we used resting platelets without staining and platelets stained with (i) anti-mouse PE-conjugated IgG1 isotype control (Beckman Coulter), for P-selectin assay; (ii) anti-mouse FITC-conjugated IgM isotype control, for PAC1 test (Thermo-Fisher, Waltham, MA, USA); and (iii) anti-mouse APC-conjugated IgG, for Annexin V-DY-634 corresponding to the allophycocyanin (APC) fluorochrome (Abcam). After incubation, PBS (400 μl) was added and the samples were analyzed using a CytoFLEX SRT flow cytometer (Beckman Coulter). At least 15,000 events were acquired. The results were analyzed using the CytExpert SRT software (Beckman Coulter). As a positive control, platelets were activated with the calcium ionophore A23187 (5 μM final concentration) (Sigma-Aldrich, St. Louis, MO, USA). After incubation, exposed PS was detected by adding 400 μl of annexin-V solution (Abcam).

### Fluorescence microscopy techniques

PRP was prepared as described above^89,90^. Isolated platelets were seeded in serum-free Iscove’s Modified Dulbecco’s medium (2 × 10^6^ platelets/well) in 24-well culture plates containing a glass coverslip coated with gelatine. After 24 h, resting platelets were incubated with increasing concentrations of αSyn-GFP (0–2 μg) in HBS. In the same experiment, resting platelets were first activated with 10 µM TRAP6 and then added with αSyn-GFP. Untreated platelets and platelets stimulated with TRAP6, without αSyn-GFP, were used as controls. The resting and activated platelets were incubated for 12 h with αSyn-GFP (0.7 and 1.4 μM), washed twice with PBS and fixed for 20 min in 2% paraformaldehyde. The slides were mounted with Mowiol antifade solution (Sigma-Aldrich, St. Louis, MO, USA) and directly observed using a DMI6000-CS fluorescence microscope (Leica Microsystem, Wetzlar, Germany). The images were acquired using a 100x/1.4 oil immersion objective magnification. The same platelet samples, incubated with αSyn-GFP, were analyzed by confocal microscopy, using a TCS SP8 microscope (Leica Microsystem). Confocal images were acquired using a 63x/1.4 oil immersion objective (image size: 1024 × 1024 pixels) and a DFC365FX camera, after applying a z-stack of 1.5 µm. The expression of endogenous (i.e. cytoplasmic) αSyn on resting and TRAP6-activated platelets was detected by immunofluorescence microscopy, using the same equipment as above, without adding αSyn-GFP. Resting or activated platelets were incubated for 1 h at 37 °C with 5 μg/ml mouse anti-human αSyn monoclonal antibody [α-Synuclein(211):sc-12767] from Santa Cruz Biotechnology (Dallas, TX, USA), followed by the addition of a diluted (1:200) goat anti-mouse IgG conjugated with Alexa Fluor 594 (Thermo-Fisher). Both primary and secondary antibodies were diluted in PBS, containing 0.5% bovine serum albumin. Unspecific binding was assessed by incubating platelets with the secondary antibody alone, in the absence of the primary antibody. The images were taken using both differential interference contrast (DIC) and fluorescence objectives and processed using the Leica Application Suite 3.1.1. software (Leica Microsystem).

### Fibrin generation assays

Fibrin generation was started by adding αT (1 nM) to a freshly desalted Fb solution (0.44 μM) in HBS at 37 °C, while the time course of clot formation was followed by continuously recording the absorbance of the solution at 350 nm (i.e. the turbidity) on a double beam V-630 Jasco (Tokyo, Japan) spectrophotometer^53,57,60^. The effect of αSyn was estimated by first incubating αT with increasing concentrations (0–20 μM) of αSyn and then adding a desalted solution of Fb. Typically, a fibrin clotting curve (i.e. the time course increase of turbidity) shows a sigmoidal shape, with (i) a lag phase, corresponding to the time necessary for the longitudinal elongation of the protofilaments; (ii) a linear rise of the turbidity signal, resulting from lateral aggregation of the protofilaments above a certain threshold length; and (iii) a plateau, when most of the protofilaments have been transformed into fibers, which then branch and assembly into the final fibrin network^49^.

### Enzymatic activity assays

The hydrolytic activity of αT was determined at 37 °C in HBS on the chromogenic substrate S2238 by measuring the release of pNA at 405 nm (ε^M^_405nm_ = 9920 M^-1^·cm^-1^), whereas the kinetics of FpA and FpB release and hydrolysis of the synthetic peptide PAR1(38–60), along with the corresponding specificity constants k_cat_/K_m_ were determined as described elsewhere^53,60^. For details, see the Supplementary Material.

### Spectroscopic methods

#### Ultraviolet absorption spectroscopy

Concentrations of protein/peptide solutions were determined by measuring the absorbance at 280 nm on a Jasco V-630 double-beam spectrophotometer, using the following molar absorptivity values (ε_M_^280^): plasma αT and β_T_T, 67.161 M^−1^·cm^−1^; recombinant rS195A, 66.424 M^−1^·cm^−1^; ProT, 99.360 M^−1^·cm^−1^; αSyn, 5.960 M^−1^·cm^−1^; 6xHis-αSyn(1–96), 1.490 M^−1^·cm^−1^; αSyn(103–140), 4.470 M^−1^·cm^−1^; hirugen, 418 M^−1^·cm^−1^; Hir(1–47), 3.355 M^−1^·cm^−1^; fibrinogen γ′–peptide, 837 M^−1^·cm^−1^; [F]-hirugen at 492 nm, 68.000 M^−1^·cm^−1^; PABA at 336 nm, 548 M^−1^·cm^−1^; S2238 at 316 nm, 12.700 M^−1^·cm^−1^. The concentration of active αT was also determined by active site titration with hirudin and was found identical (± 5%) to that determined spectrophotometrically.

#### Fluorescence spectroscopy

Binding measurements were carried out at 37 °C in HBS, containing 0.1% PEG-8000 (w/v), on a Jasco FP-6500 spectrofluorimeter. Aliquots (2–10 μl) of αSyn or αSyn(103–140) in HBS were added, under gentle magnetic stirring, to a αT solution (70 nM) in the same buffer. At each ligand concentration, the samples were incubated for 2 min at 37 °C and excited at 295 nm, using an excitation/emission slit of 5/10 nm. The fluorescence intensity was recorded at 334 nm, i.e. the λ_max_ of αT emission, after subtracting the corresponding spectra of the ligands alone. Fluorescence data were corrected for sample dilution (< 5%). To prevent photobleaching of Trp residues, a 1-cm pathlength quartz cuvette (2 ml) with two frosted walls was used, diffusing the incident light inside the sample. The optical density of the solution was always kept < 0.05 units at both λ_ex_ and λ_em_, to avoid the inner filter effect^59^. A similar procedure was used to measure the affinity of all other site-specific ligands tested in this work (i.e. PABA, S2238, Hir(1–47), hirugen, [F]-hirugen and fibrinogen γ′–peptide) for αT in the presence of constant, saturating concentration of αSyn or αSyn(103–140) (20 μM). When the binding of PABA was studied, samples were excited at 336 nm and the emission of PABA was recorded at 375 nm, after baseline subtraction and correction for inner filter effect, as detailed elsewhere^59^. For binding of [F]-hirugen, aliquots of αT S195A mutant stock solution (30 μM) were incrementally added to a [F]-hirugen solution (60 nM). The samples were excited at 492 nm and the decrease in fluorescence intensity of [F]-hirugen was recorded at 516 nm as a function of αT^58^. Analysis of the binding data was performed as earlier described^59^ and detailed in the Supplementary Material.

#### Surface plasmon resonance

SPR analyses were performed on a Biacore X-100 dual flow-cell instrument from GE Healthcare. 6xHis-αSyn was immobilized noncovalently on a Ni^2+^-chelated nitrilotriacetate (NTA) carboxymethyldestrane sensor chip and incremental concentrations of S195A were loaded. The Ni^2+^-NTA/6xHis-αSyn chip assembly was prepared as follows: the NTA chip (GE Healthcare) was first washed (flow-rate: 30 μl/min) with 0.35 M EDTA, pH 8.3 (contact time: 700 s) and then loaded with 0.5 mM NiCl_2_ solution (contact time: 400 s); excess Ni^2+^ was removed by injecting 3 mM EDTA solution (contact time: 350 s), whereas non-chelating NTA-groups were irreversibly blocked with ethanolamine, after carboxylate activation (contact time: 800 s) with N-(3-dimethylaminopropyl)-N′-ethylcarbodiimide and N-hydroxysuccinimide; finally, a solution of 6xHis-αSyn (200 nM) was injected on the sensor chip (contact time: 400 s) to yield a final immobilization level of 2194 response units (RU). The Ni^2+^-NTA/6xHis-αSyn sensor chip was challenged (flow-rate: 30 μl/min; contact time: 350 s) with increasing concentrations of inactive S195A thrombin mutant, β_T_T, and ProT. All measurements were carried out at 37 °C in HBS-EP^+^ buffer (10 mM HEPES, pH 7.4, 0.15 M NaCl, 50 μM EDTA, 0.005% v/v polyoxyethylene sorbitan). Between two consecutive runs, the regeneration of Ni^2+^-NTA/6xHis-αSyn chip was achieved with HBS-EP^+^ buffer, containing 2 M NaCl. Each sensogram was subtracted for the corresponding baseline, obtained on the reference flow cell and accounting for nonspecific binding, i.e. typically less than 2% of RU_max_. The binding data were analyzed using the BIAevaluation software, as detailed in the Supplementary Material^53,60^.

### Computational methods

Electrostatic potential calculations were performed using APBS software^92^. The coordinates of human PAR1 (3vw7)^81^ and P2Y_12_ receptor (4ntj)^82^ bound to the inhibitors Vorapaxar and AZD1283, respectively, were considered for calculations. PAR4 (UniProt code: Q96RI0; amino acid residues Asp65-Phe347) structure was modeled by homology on the template structure of PAR1 (PDB code: 3vw7; UniProt code: P25116; amino acid residues Asp91-Cys378)^81^ with which it shares 34.6% sequence identity and 56.1% sequence similarity. The Swiss-Model software was used^93^. For αT, calculations were run on the non-glycosylated X-ray structure of αT (1 ppb), after removal of the coordinates of the inhibitor D-Phe-Pip-Arg-chloromethylketone, water and HEPES molecules^64^. The electrostatic contribution of Na^+^-ion bound to PAR1 was not considered in our calculations. A solvent dielectric constant of 78.14 and a protein dielectric constant of 2.0 at 310 K in 150 mM NaCl were used. Final electrostatic maps were constructed by subtracting the protein self-energies from the calculated map using the DXMATH utility in APBS. Notably, to facilitate crystallogenesis, T4 lysozyme (T4L) and the BRIL domain were inserted into the intracellular loop 3 of PAR1^81^ and P2Y_12_R^82^, respectively. In the recombinant PAR1-T4L fusion protein the N-terminal exodomain was missing. The coordinates of the bound inhibitor were virtually removed, along with the inserted structure of T4L and BRIL. To minimize artefactual charge perturbations, following virtual domain excision, the remaining N- and C-termini were made neutral by acetylation or amidation.

Exogenous (i.e. plasmatic) or endogenous (i.e. internal) platelet αSyn concentrations were estimated using the following assumptions and semplifications. Platelets counts in WB: 150.000–450.000/μl, mean in WB ~ 300.000/μl, mean in PRP ~ 600.000/μl. In normal subjects, > 95% platelets have a round/oval shape, with a diameter (d) of 1.5–3 μm (mean d = 2.25 ± 0.75 μm) and a volume of 7.5–12 fl, mean ~ 9.7 fl (1 fl = 1·10^–15^ lt)^86^. Recent data demonstrate that after activation by αT, the cross-sectional area of platelets is halved, from 7.5 μm^2^ in resting platelets to 3.5 μm^2^ in activated platelets^84^, with an estimated ~ threefold reduction in the mean platelet volume, from 9.7 to 3.2 fl. Hence, the total volume of activated platelets in 1 μl of PRP is 600.000 × 3.2 fl = 1.92 nl. The concentration of αSyn in PRP can be considered the same as that experimentally determined in the plasma 25.4 ± 9.3 ng/ml (1.8 nM)^17^. Assuming that all free αSyn (1.8 fmoles) contained in 1 μl of PRP quantitatively binds to the membrane surface of activated platelets, delimiting a volume of 1.92 nl, the resulting “local” apparent αSyn concentration is 0.93 μM. For “internal” platelet αSyn, Burkhart et al. determined that on average a single platelet (3.2 fl) contains 38,100 ± 3430 αSyn molecules (6.33·10^–20^ mol)^20^. Assuming that, after activation, all internal αSyn is expressed on the platelet membrane, an apparent [αSyn] of 19.8 μM is obtained. Likewise, the average number of PAR1 molecules *per* resting platelet has been determined by flow cytometry as 1276 ± 320 (n = 70)^88^, yielding a platelet PAR1 apparent concentration of 0.22 ± 0.05 μM.

### Statistical analysis

For flow cytometry and (immune)fluorescence microscopy measurements, statistical analysis was performed using GraphPad Prism v5. One-way ANOVA and Student’s t-test applications were used, while results were considered significant (*) when *p* < 0.05.

## Supplementary Information

Supplementary Information.

## Data Availability

All other data that support the findings of this study are available from the corresponding author upon reasonable request.
