# Supplementary material for: Exogenous human α-Synuclein acts in vitro as a mild platelet antiaggregant inhibiting α-thrombin-induced platelet activation
Source: Sci Rep. 2022 Jun 14;12:9880. doi: 10.1038/s41598-022-12886-y (PMC9198058; doi:10.1038/s41598-022-12886-y)
Supplement: Supplementary file 1 — Supplementary Information. [file 41598_2022_12886_MOESM1_ESM.docx]

**SUPPLEMENTARY MATERIALS**

#### Exogenous human α-synuclein acts *in vitro* as a mild platelet antiaggregant inhibiting α-thrombin-induced platelet activation

Laura Acquasaliente ^1, +^, Giulia Pontarollo ^1, +, b^, Claudia Maria Radu ^2, 3, +^, Daniele Peterle ^1, c^, Ilaria Artusi ^1^, Anna Pagotto ^1^, Federico Uliana ^1, d^, Alessandro Negro ^4,^ *, Paolo Simioni ^3,^ *, Vincenzo De Filippis ^1, 5^ *

^1^ Laboratory of Protein Chemistry and Molecular Hematology, Department of Pharmaceutical and Pharmacological Sciences, University of Padua, Padua, Italy

^2^ Department of Women’s & Children’s Health, University of Padua, Padua, Italy

^3^ Thrombotic and Hemorrhagic Diseases Unit, Department of Medicine, University of Padua, Padua, Italy

^4^ Department of Biomedical Sciences, University of Padua, Padua, Italy

^5^ CRIBI, Biotechnology Center, University of Padua, Padua, Italy

**Corresponding*: Vincenzo De Filippis, Department of Pharmaceutical and Pharmacological Sciences, School of Medicine, University of Padua, via Marzolo, 5, Padua, 35131 Italy. Phone: (+39) 0498275698, e-mail: [vincenzo.defilippis@unipd.it](mailto:vincenzo.defilippis@unipd.it). Alessandro Negro, Dept. of Biomedical Sciences, University of Padua, viale G. Colombo 3, I-35100 Padua, Italy. Phone: (+39) 0498276166, e-mail: [alessandro.negro@unipd.it](mailto:alessandro.negro@unipd.it). Paolo Simioni, Thrombotic and Hemorrhagic Diseases Unit, Department of Medicine, University of Padua, Padua, via Giustiniani, 2, 35128 Padova, Italy. Phone: (+39) 0498212667, e-mail: paolo.simioni@unipd.it

^+^ These authors contributed equally to this work.

*Author information*:

^b^ Present address: Center for Thrombosis and Hemostasis (CTH) University Medical Center Mainz, Langenbeckstraße 1, 55131 Mainz, Germany

^c^ Present address: Dept. of Chemistry and Chemical Biology, Northeastern University, 360 Huntington Ave. 02115, Boston, MA, USA

^d^ Present address: Institute of Molecular Systems Biology, ETH Zurich , 8093 Zurich, Switzerland.

*Running title*: α-Synuclein inhibits thrombin-induced platelet aggregation

*Keywords*: α-synuclein, thrombin, platelets, coagulation, Parkinson’s disease.

**Extended Methods**

*Production and characterization of recombinant αSyn derivatives*

All recombinant human synuclein derivatives (i.e. αSyn, 6xHis-αSyn, 6xHis-αSyn(1-96), and αSyn-GFP), were produced and purified as previously detailed ^1,2^. Briefly, BL21*(DE3) *Escherichia coli* pLysS cells were transformed, using the heat-shock method, with *p*RSET-B plasmid containing the human αSyn gene and selected in Luria-Bertani (LB) Agar Amp^+^ (0.1 mg/ml) solid culture medium overnight. The transformed cells were grown at 37°C in LB broth Amp^+^ (0.05 mg/ml) and induced (OD = 0.6) with isopropyl β-D-1-thiogalactopyranoside (IPTG, 0.1 mg/ml) under vigorous shaking. For αSyn and αSyn-GFP, after 3 h of IPTG induction, bacteria were harvested by centrifugation (6.000 rpm, 15 min, at 4°C), and the pellet was sonicated in 40 mM Tris-HCl, pH 8.0, 0.1 M NaCl (buffer A). After 10-min boiling, the suspension was centrifuged (12,000 rpm, 10 min, 4°C). The supernatant, containing soluble αSyn or αSyn-GFP, was dialyzed overnight at 4°C against buffer A, containing 2 mM EDTA. For 6xHis-αSyn and 6xHis-αSyn(1-96), after sonication in buffer A, the recombinant proteins were purified by IMAC. Bacterial lysis supernatant (50 ml) was loaded onto a fast-flow Ni^2+^-IMAC (1 x 3 cm) HiTrap column, using a model P-1 peristaltic pump (Pharmacia, Uppsala, Sweden) at a flow rate of 0.1 ml/min. The flow-through was discarded and the column connected to an Äkta-purifier system (Marlborough, MA, USA). After washing with buffer A (60 ml), 6xHis-tagged proteins were eluted from the column (0.5 ml/min) with buffer A, pH 6.5, containing 0.4 M imidazole. The material eluted in correspondence of the major chromatographic peak was collected and dialyzed overnight at 4°C against phosphate buffered saline, pH 7.4. Recombinant proteins were further purified by RP-HPLC on a semipreparative column C18 (10 x 250 mm, 5μm, 300Å) from Grace-Vydac (Hesperia, CA, USA), eluted with a linear acetonitrile-0.078% trifluoroacetic acid gradient at a flow rate of 1.5 ml/min. After lyophilization, recombinant proteins in water:acetonitrile (1:1 v/v), containing 1% formic acid, were characterized by high-resolution mass spectrometry using a Waters (Milford, MA, USA) Xevo-G2S Q-TOF spectrometer. To obtain purified αSyn in the monomeric state, the lyophilized protein (1 mg) was dissolved in 2 mM NaOH (100 μl) and 1M NaOH (10 μl), up to pH 11.0. After centrifugation (15,000 rpm, 15 min), the supernatant was removed and added with 0.1 M Tris-HCl, pH 7.0 (200 μl), down to pH 8.0. Freshly dissolved αSyn samples were used for further spectroscopic and functional analyses. The purified αSyn solutions were divided into aliquots, lyophilized, and stored at -20°C. After thawing in an ice-water bath, αSyn aliquots were immediately used for subsequent functional/binding analyses.

*Enzymatic activity assays*

Briefly, human fibrinogen (Fb) (ε^M^_280nm_ = 5.1·10^5^ M^-1^·cm^-1^) was desalted on an in-house packed (8x125mm) G10 fast-flow column (GE Healthcare, Chicago, IL, USA) eluted with HBS, pH 7.4, at a flow-rate of 0.3 ml/min. Freshly prepared Fb (0.35 μM) was reacted at 37°C with human αT (300 pM) in the presence of 15 μM αSyn and at fixed time points proteolysis mixtures were added with formic acid (2% v/v final concentration) to block the proteolysis reaction and induce precipitation of unreacted Fb. After centrifugation (10.000 g, 5 min, 4°C), the supernatant (1.0 ml) was removed, lyophilized, dissolved in a 6 M guanidinium hydrochloride solution (170 μl) and injected (100 μl) into a RP-HPLC (4.6 x 250 mm) C18 column (Grace-Vydac, Columbia, MD, USA). The column was equilibrated with 40 mM ammonium phosphate buffer, pH 3.1, and eluted with an acetonitrile gradient. The absorbance of the effluent was recorded at 205 nm and the amount of released FpA (ε^M^_205nm_ = 4.40·10^4^ M^-1^·cm^-1^) and FpB (ε^M^_205nm_ = 5.12·10^4^ M^-1^·cm^-1^) was determined by integrating the area under the chromatographic peaks. A LC-4000 HPLC system (Jasco, Tokyo, Japan) was used for all analyses.

Specificity constants, k_cat_/K_m_, for fibrinopeptide release of were determined by interpolating the data points to **equations 1** and **2** ^3,4^:

$\text{[FpA]}_{\text{t}}=\text{[FpA]}_{\infty}\cdot(1-\text{e}^{\text{-k't}})$ (**eq. 1**)

$\text{[FpB]}_{\text{t}}=\text{[FpB]}_{\infty}\cdot(1+\alpha\cdot\text{e}^{\text{-k't}}-\beta\cdot e^{-k''t})$ (**eq. 2**)

where [FpA]_t_ or [FpB]_t_ and [FpA]_∞_ or [FpB]_∞_ are the concentration of FpA or FpB at time t and ∞, respectively, and k′ and k′′ are the observed kinetic constants for the release of FpA or FpB, obtained as fitting parameters. Under pseudofirst-order conditions and low substrate concentration, the specificity constants could easily be determined as k_catA_/K_mA_ = k′/[E] and k_catB_/K_mB_ = k′′/[E], where [E] is the protease concentration.

Hydrolysis of the synthetic peptide PAR1(38-60) (1 µM) by αT (150 pM) was carried out at 25°C in TBS, in the presence of αSyn (15 μM). At time points, aliquots (360 μl) were taken, acid quenched (10 μl, 4% aqueous TFA) and loaded (350 µL) onto a Grace-Vydac (4.6 x 250 mm) C18 column. The column was eluted with a linear acetonitrile-0.078% TFA gradient from 10-45% in 40 min and the release of PAR1(42-60) (ε^M^_205nm_ = 95870 M^-1^·cm^-1^) was quantified by integrating the area under the chromatographic peak. The kinetic data were interpolated with **equation 3**, describing a pseudo-first order reaction ^3-5^:

$\text{[P]}_{\text{t}}=\text{[P]}_{\infty}\cdot[1-\exp(-k{}_{obs}\cdot t)]$ (**eq. 3**)

where [P]_∞_ is the concentration of the fragment PAR1(42-60) when the proteolysis reaction was complete and k_obs_ is the observed kinetic constant for PAR1(38-60) hydrolysis, obtained as a fitting parameter. Regarding the release of fibrinopeptides, under pseudofirst-order conditions and low substrate concentration, k_cat_/K_m_ could be derived as k_obs_/[E]. Notably, PAR1(38-60) reproduces the substrate binding properties of the extracellular PAR1 domain in platelets, as it contains both the exosite-1 binding sequence for αT and the scissile bond Arg^41^-Ser^42^.

The difference in free energy change of binding of a substrate (ΔΔG_b_*) to the enzyme active site in the transition state, in the absence (-) and presence (+) of αSyn, is given by equation **4**:

**eq. 4**

where R is the gas constant (1.987 cal/(mol·K), T is the absolute temperature (K), and s is the specificity constant (s = k_cat_/K_m_) of the enzyme-catalysed reaction. The effect (either positive or negative) of αSyn on αT catalysis is taken as significative when ΔΔG_b_* is greater, in absolute value, than the internal energy (E) of the system at a given temperature: E = R·T ^6,7^.

*Dynamic light scattering*

Measurements were performed at 37°C on a Zetasizer-Nano-S instrument (Malvern Instruments, Worchestershire, UK) at a fixed angle (i.e. 173°) from the incident light (i.e. He–Ne 4 mW laser source at 633 nm). Polystyrene cuvettes (1-cm path length, 100 μl) (Hellma, Switzerland) were used for all measurements. Each measurement consisted of a single run (15 s). Scattering data were analyzed with Nano-6.20 software and expressed as percentage of volume size distribution, from which the value of *d_H_* and %PD were extracted ^5^, where *d_H_* is the diameter of a hard sphere that diffuses at the same speed as the molecule being measured, and %PD is the width of the particle size distribution of a protein in a given sample.

*Fluorescence spectroscopy.*

The data points were interpolated with **equation 5**, describing the single-site binding model R + L ↔ RL ^8^:

$\Delta\text{F}=\Delta\text{F}_{\text{max}}\text{ }\cdot\text{ }\frac{\text{[L]}}{\text{K}_{\text{d}}+\text{[L]}}$ (**eq. 5**)

where L is the concentration of the ligand, ΔF and ΔF_max_ are the changes in fluorescence intensity measured at intermediate or saturating ligand concentrations, while the dissociation constant, K_d_, was obtained as a fitting parameter. For Hir(1-47) and [F]-hirugen binding to αT, fluorescence data were interpolated with **equation 6,** describing the tight-binding model ^8^.

$\Delta F=\Delta F_{\max}\cdot\frac{b+\sqrt{b^{2}-4[R][L]}}{2[R]}$ (**eq. 6)**

$$b=([R]+[L]+K_{d})$$

where [R] and [L] are the total enzyme or ligand concentrations.

The difference in free energy change of binding of a ligand/inhibitor (ΔΔG_b_) to αT, in the absence (-) and presence (+) of αSyn, is given by **equation 7**:

(**eq. 7)**

where R is the gas constant (1.987 cal/(mol·K), T is the absolute temperature (K), K_d_ is the dissociation constant of thrombin-ligand complex. The effect (either positive or negative) of αSyn on αT binding is taken as significative when ΔΔG_b_ is greater, in absolute value, than the internal energy (E) of the system at a given temperature: E = R·T ^6,7^.

*Surface plasmon resonance*

The dissociation constant (K_d_) relative to the binding of αT to immobilized αSyn was obtained as a fitting parameter by plotting the RU value at the steady state (RU_eq_) *versus* [αT] and interpolating the data points with **equation** **8**, describing 1:1 binding model:

$\text{RU}_{\text{eq}}=\text{RU}_{\text{max}}\cdot\frac{\text{[L]}}{\text{K}_{\text{d}}+\text{[L]}}$ (**eq. 8**)

where L is the concentration of αT, while RU_eq_ and RU_max_ are the RU values measured (at the steady state) with intermediate or saturating [L] ^3,4^.

*Isothermal titration calorimetry* (ITC)

ITC titrations were performed at 25±0.1°C in 20 mM HEPES pH 7.4, 0.15M, using a MicroCal VP-ITC instrument, as described ^9^. To a S195A thrombin mutant solution (1.7 ml, 2 µM) were sequentially added 25 aliquots (10 μl each) of αSyn stock solution (40 μM), under continuous stirring (307 r.p.m.) and a delay of 4 min after each injection. Before analysis, protein samples were dialyzed overnight in the same buffer, using a Slide-A-Lyzer (3.5-kDa cut-off) from ThermoFischer Scientific (Waltham, MA, USA), and thoroughly degassed. The heat of dilution was determined in control experiments by injecting aliquots (10 µl) of αSyn stock solution (40 µM) into buffer and this was subtracted from the integrated binding isotherm prior to curve fitting. The thermograms were analysed using the MicroCal ITC Data Analysis software.

**Extended Results**

For the wild-type αSyn and αSyn-GFP protein mutant, the bacterial pellet was sonicated and then boiled for 10 min. After centrifugation, the supernatant enriched with αSyn or αSyn-GFP was dialyzed and further purified by RP-HPLC. At variance, 6xHis-αSyn and 6xHis-αSyn(1-96) were purified by immobilized metal ion affinity chromatography (IMAC), followed by RP-HPLC. The purity of αSyn species was checked by SDS-PAGE and RP-HPLC (>98%), while their chemical identity was established by high-resolution mass spectrometry, which was found in agreement with the protein amino acid composition within 20 ppm mass accuracy (**Supplementary Table S1 and Figure S1**). The monomerization of purified αSyn was achieved by alkaline treatment, i.e. dissolution of αSyn lyophilizate with NaOH solution, at pH 11.0, followed by the addition of 0.1 M Tris-HCl, pH 7.0, down to pH 8.0 ^10^ (see below).

*Monomerization of recombinant aSyn*

With the aim to obtain highly monomeric αSyn preparations for subsequent spectroscopic and functional analysis, different conditions were explored, including i) TBS [5 mM Tris-HCl, pH 8.0, 0.2 M NaCl, 0.1% PEG-8000 (v/w)], ii) TBS containing 7% (v/v) DMSO, iii) TBS containing 5 M Gnd-HCl, or iv) 50 mM Tris-HCl, pH 8.0, after alkaline treatment. Notably, the alkaline treatment consisted in the dissolution of αSyn lyophilizate with NaOH solution, at pH 11.0, followed by addition of an equal volume of 0.1 M Tris-HCl, pH 7.0, down to pH 8.0.

The presence of protein aggregates was estimated from the shape of UV-absorption spectra (**Supplementary Fig. 1B**), i.e. the ratio of the absorbance values at 275 and 250 nm (r = A_275nm_/A_250nm_), and from the relative intensity of the apparent fluorescence emission of αSyn solutions (F/F_0_) at increasing protein concentrations (**Supplementary Fig. 1C**). The A_275nm_/A_250nm_ ratio is a sensitive measure of protein aggregation, as the intensity of the scattered light exponentially increases at lower wavelengths. Likewise, the slope of the straight line of F/F_0_ vs. [αSyn] is a signature of the presence of protein aggregates that more intensely scatter light, which is then recorded as “apparent” fluorescence emission. UV-absorption and fluorescence spectra of αSyn solutions were compared with those of a model compound solution, 2:1 (mol:mol) N^α^-acetyl-Tyr-NH_2_ / N^α^-acetyl-Phe-NH_2_ solution, which was taken as a model of Tyr and Phe spectroscopic properties in αSyn monomeric state.

UV-absorption spectra (**Supplementary Fig. 1B**) of αSyn show that in TBS alone the r = 1.6, is markedly lower than that estimated for the model compound solution (r = 3.1), suggesting the presence of protein aggregates. Addition of 5 M Gnd-HCl or 7% DMSO to TBS, or alkaline treatment progressively increased r values (r = 3.0) up to those of model compound solution. Likewise, fluorescence data (**Supplementary Fig. 1C)** show that only alkaline treatment proved effective in decreasing the slope (m) of the interpolating straight line in the plot of F/F_0_ vs. [αSyn], measured in TBS alone (m = 0.2), to a value identical to that obtained for the model compound solution (m = 0.02).

The monomeric state of αSyn preparation after alkaline treatment was confirmed by Dynamic Light Scattering (DLS) measurements, from which the hydrodynamic diameter (d_H_) and the percent polydispersity (%PD) were extracted (**Supplementary Fig. 1D**). Notably, d_H_ is the diameter of a hard sphere that diffuses at the same speed as the molecule being measured, while %PD is a parameter describing the width of the particle size distribution of a protein in a given sample. In DLS analysis, the time-dependent fluctuations of scattered light from molecules of different size in solution is measured and from the rate of these fluctuations the translational diffusion coefficient (*D*) is determined. The value of *d_H_* is then derived from the Stokes-Einstein equation, *d_H_ = 2·kT/6πηD*, where *k* is the Boltzmann constant, *T* is the absolute temperature and *η* is the solution viscosity. The monomeric state of the αSyn preparation was confirmed by dynamic light scattering measurements (DLS), from which a hydrodynamic diameter (d_H_) of 56 ± 6 Å was estimated, with a percent polydispersity (%PD) as low as 11.6% (**Supplementary Figure S1**), indicative of a monodispersed protein solution. Notably, the size of αSyn reported in this study is lower than that predicted for a fully unfolded protein of 140 amino acids such as αSyn (d_H_^U^ = 68 Å), but still compares favorably with that determined experimentally by small-angle X-ray scattering (d_H_ = 54 ± 2 Å) and size-exclusion chromatography (d_H_ = 55 ± 6 Å) ^10^, and is in agreement with the loosely packed dynamic structure recently elucidated for monomeric αSyn ^11^.

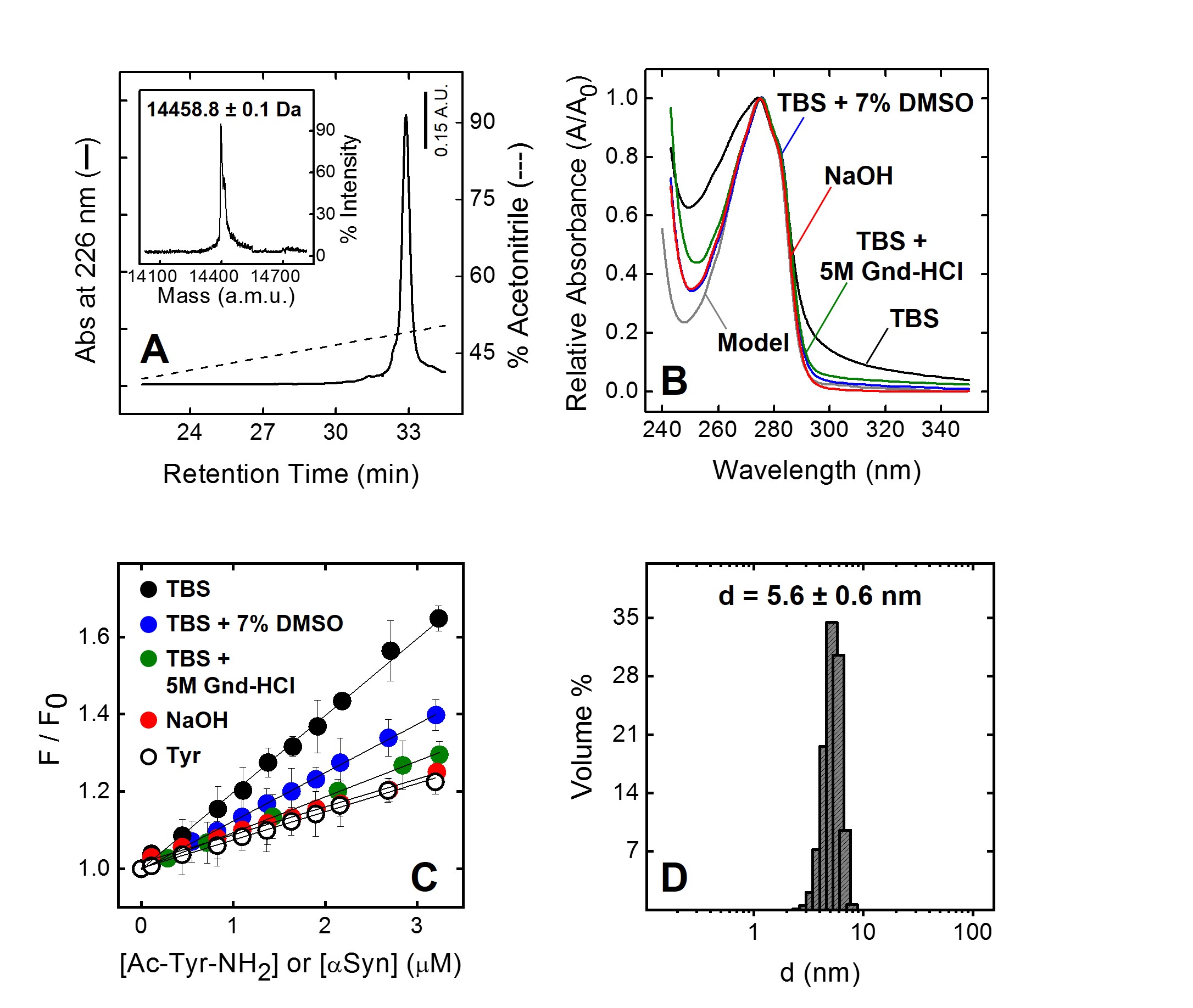


**Supplementary Figure S1. Purification and Characterization of monomeric αSyn.** (**A**) RP-HPLC analysis of purified recombinant αSyn, using a semiprepartive (1x25 cm) C18 column eluted with a linear 0.078% TFA-acetonitrile gradient (---). (**Inset**) MS analysis of RP-HPLC purified αSyn. (**B**) Near-UV absorption spectra of αSyn (130 μM) in different solvents (**▬**) TBS: 5mM Tris-HCl, pH 8.0, 0.2 M NaCl, 0.1% PEG-8000 (v/w); (**▬**) TBS-DMSO: TBS containing 7% (v/v) DMSO; (**▬**) TBS-Gnd: TBS containing 5 M Gnd-HCl; (**▬**) NaOH: αSyn was first dissolved in 100 μl of 2 mM NaOH and 10 μl of 1 M NaOH and then added with 200 μl of 0.1 M Tris-HCl pH 7.0 (see Methods). UV-absorption spectra of αSyn samples at 25°C. The nominal αSyn concentration was the same under all experimental conditions and the spectra were normalized (A/A_0_) for the absorbance intensity of αSyn in TBS (A_0_) at the λ_max_ (275 nm). As a control, the spectrum of Ac-Tyr-NH_2_ and Ac-Phe-NH_2_, mixed in the same molar ratio (2:1) as that present in αSyn (**Model**), is also reported (**▬**). (**C**) Concentration-dependence of αSyn fluorescence intensity recorded at 25°C under different solvent conditions, as reported above. αSyn samples were excited at 280nm and the fluorescence signal was recorded at the λ_max_ (303nm). The data are expressed as F/F_0_ ratio, where F and F_0_ is the fluorescence signal of the buffer solvent in the presence or absence of increasing [αSyn]. For comparison, the data of Ac-Tyr-NH_2_ (Tyr, ○) emission in TBS are also included. Linear interpolation of the fluorescence data yielded the following slope values: TBS, 0.201 ± 0.004; TBS-DMSO, 0.125 ± 0.001; TBS-Gnd, 0.092 ± 0.001; NaOH, 0.022 ± 0.001; Tyr, 0.073 ± 0.001. (**D**) DLS analysis at 37°C of purified αSyn (50 μM) after alkaline treatment, as described above. The data are expressed as the volume size distribution and *d* is the average molecular diameter.

**Supplementary Figure S2. Proteolysis of recombinant** **αSyn (A) and 6xHis-αSyn (B) by αT.** RP-HPLC analysis of the proteolysis reaction of αSyn and 6xHis-αSyn (5 μM) with αT (72 nM), carried out for 4 hours at r.t. in TBS, pH 7.4. An aliquot (300 μl) of the proteolysis reactions was loaded onto a Grace-Vydac C18 (4.6 x 150 mm) column, eluted with a linear acetonitrile/0.1%-TFA gradient (---) at a constant flow rate (0.8 ml/min). The absorbance of the effluent was recorded at 220 nm. The material eluted with the chromatographic peaks was collected and analysed by high-resolution mass spectrometry. The labels near the chromatographic peaks refer to the proteolysis products of αSyn and 6xHis-αSyn. The data clearly indicate that, under the experimental condition used in this work, αT can cleave 6xHis-αSyn at Lys^6^-Gly^7^ peptide bond, whereas underivatized αSyn is fully resistant to αT proteolysis.


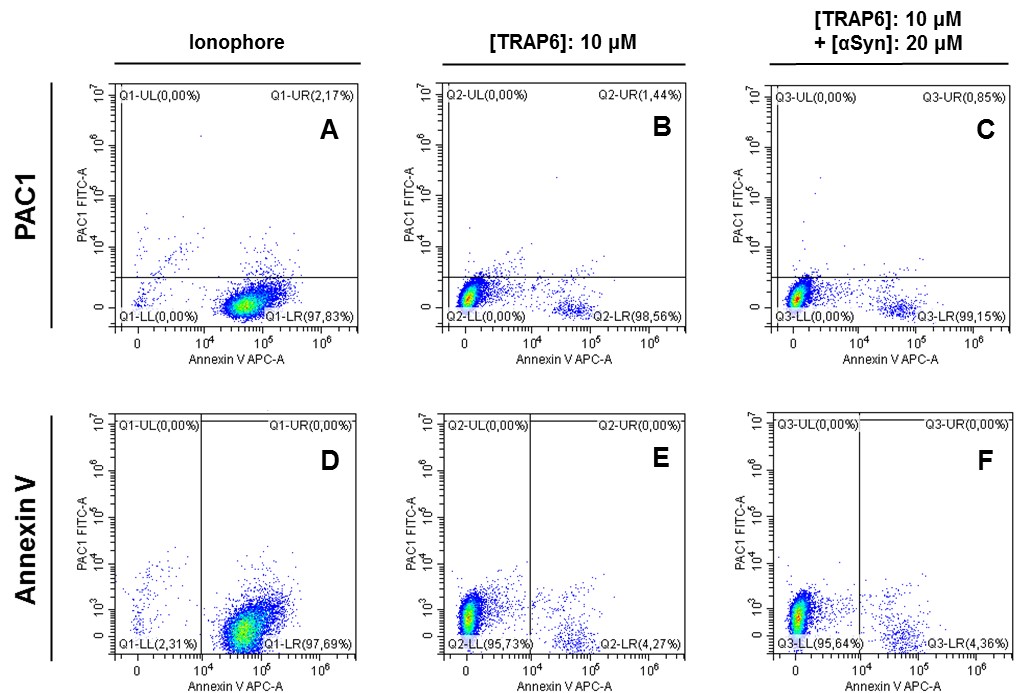


**Supplementary Figure S3.** Representative flow cytometry dot plots of the total per cent expression of activated GpIIb/IIIa and phosphatidylserine (PS), as given by PAC1 (panels **A**, **B**, **C**) and annexin-V (panels **D**, **E**, **F**)) assays (n = 3), respectively, after activation with the ionophore A23187 (5 μM) (**A**, **D**), TRAP6 alone (10 μM) (**B**, **E**) and TRAP6 (10 μM) (**C**, **F**) in the presence of αSyn (20 μM). Vertical and horizontal lines indicate the gating strategy. Q1-UR, Q2-UR and Q3-UR values indicate the total % expression of activated GpIIb/IIIa (PAC1) under different activation conditions. Q1-LR, Q2-LR and Q3-LR values indicate the total % expression of PS (Annexin V) under different activation conditions. For details, see Materials and Methods.


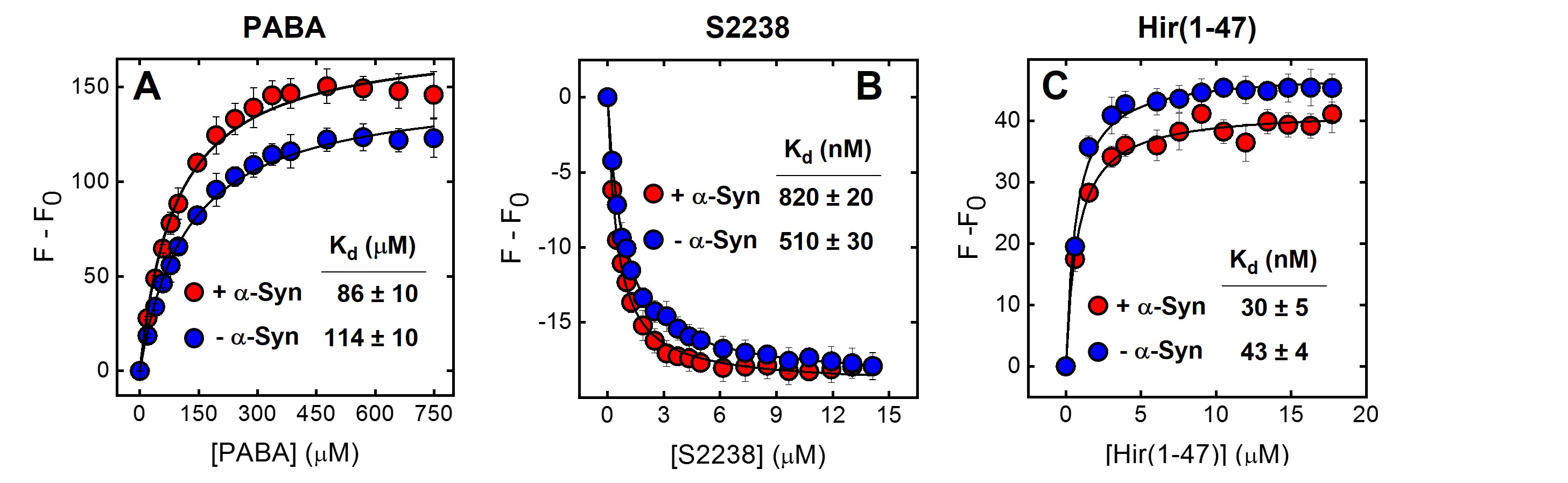


**Supplementary Figure S4. Probing the role of thrombin active site in αSyn-αT interaction**. Effect of αSyn on the affinity of the active-site ligands PABA (**A**), S2238 (**B**), or Hir(1-47) (**C**) for thrombin. Fluorescence binding measurements were carried out in HBS at 37°C by adding increasing ligand concentrations to thrombin solutions, in the absence or presence of 20 μM αSyn. For the binding of PABA, samples (40 nM) were excited at 336nm, the fluorescence intensity of the ligand was recorded at 375 nm and corrected for inner filter effect. With S2238 and Hir(1-47), protein samples (50 nM and 70 nM, respectively) were excited at 295 nm, while thrombin fluorescence was recorded at 334 nm. When the binding of S2238 to thrombin was being studied, the inactive S195A mutant was used. The data points relative to the binding of PABA and S2338 were interpolated with **eq. 5**, describing a single-site interaction model, while the data for the binding of Hir(1-47) were fitted with **eq. 6**, describing a tight-binding model. After interpolation, the K_d_ values were obtained as fitting parameters, as indicated.

**References**

1 Negro, A., Brunati, A. M., Donella-Deana, A., Massimino, M. L. & Pinna, L. A. Multiple phosphorylation of alpha-synuclein by protein tyrosine kinase Syk prevents eosin-induced aggregation. *Faseb j* **16**, 210-212, doi:10.1096/fj.01-0517fje (2002).

2 Albani, D. *et al.* Protective effect of TAT-delivered alpha-synuclein: relevance of the C-terminal domain and involvement of HSP70. *Faseb j* **18**, 1713-1715, doi:10.1096/fj.04-1621fje (2004).

3 Pontarollo, G. *et al.* Non-canonical proteolytic activation of human prothrombin by subtilisin from Bacillus subtilis may shift the procoagulant-anticoagulant equilibrium toward thrombosis. *J Biol Chem* **292**, 15161-15179, doi:10.1074/jbc.M117.795245 (2017).

4 Acquasaliente, L. *et al.* Molecular mapping of alpha-thrombin (alphaT)/beta2-glycoprotein I (beta2GpI) interaction reveals how beta2GpI affects alphaT functions. *Biochem J* **473**, 4629-4650, doi:10.1042/bcj20160603 (2016).

5 Sokolov, A. V. *et al.* Thrombin inhibits the anti-myeloperoxidase and ferroxidase functions of ceruloplasmin: relevance in rheumatoid arthritis. *Free Radic Biol Med* **86**, 279-294, doi:10.1016/j.freeradbiomed.2015.05.016 (2015).

6 Copeland, A. R. *Enzymes. A practical introduction to structure, mechanism, and data analysis*. Second edn, 122-123 (2000).

7 De Filippis, V., Quarzago, D., Vindigni, A., Di Cera, E. & Fontana, A. Synthesis and characterization of more potent analogues of hirudin fragment 1-47 containing non-natural amino acids. *Biochemistry* **37**, 13507-13515, doi:10.1021/bi980717n (1998).

8 Pozzi, N. *et al.* Loop Electrostatics Asymmetry Modulates the Preexisting Conformational Equilibrium in Thrombin. *Biochemistry* **55**, 3984-3994, doi:10.1021/acs.biochem.6b00385 (2016).

9 Ricatti, J. *et al.* Effects of point mutations in the binding pocket of the mouse major urinary protein MUP20 on ligand affinity and specificity. *Sci Rep* **9**, 300, doi:10.1038/s41598-018-36391-3 (2019).

10 Uversky, V. N., Li, J. & Fink, A. L. Evidence for a partially folded intermediate in alpha-synuclein fibril formation. *J Biol Chem* **276**, 10737-10744, doi:10.1074/jbc.M010907200 (2001).

11 Theillet, F. X. *et al.* Structural disorder of monomeric α-synuclein persists in mammalian cells. *Nature* **530**, 45-50, doi:10.1038/nature16531 (2016).
